# Devaluation underlies gendered US faculty departures across disciplines and institutions

**DOI:** 10.1093/pnasnexus/pgag306

**Published:** 2026-10-13

**Authors:** Katie Spoon, Joanna Mendy, Maria Martinez, Mirta Galesic, Daniel B Larremore, Aaron Clauset, Lauren A Rivera

**Affiliations:** Department of Computer Science, University of Colorado Boulder, Boulder, CO 80309, USA; School of Education, University of Colorado Boulder, Boulder, CO 80309, USA; Department of Information Science, University of Colorado Boulder, Boulder, CO 80309, USA; School of Education, University of Colorado Boulder, Boulder, CO 80309, USA; Santa Fe Institute, Santa Fe, NM 87501, USA; Vermont Complex Systems Center, University of Vermont, Burlington, VT 05405, USA; Complexity Science Hub, Vienna, Austria; Department of Computer Science, University of Colorado Boulder, Boulder, CO 80309, USA; Santa Fe Institute, Santa Fe, NM 87501, USA; BioFrontiers Institute, University of Colorado Boulder, Boulder, CO 80309, USA; Department of Computer Science, University of Colorado Boulder, Boulder, CO 80309, USA; Santa Fe Institute, Santa Fe, NM 87501, USA; BioFrontiers Institute, University of Colorado Boulder, Boulder, CO 80309, USA; Kellogg School of Management, Northwestern University, Evanston, IL 60208, USA

**Keywords:** computational social science, gender, higher education, faculty retention, science of science

## Abstract

Women faculty experience academia differently from men in many ways, which can lead them to consider leaving their positions. Using a large-scale survey of both current and former tenure-line faculty across nearly all US PhD-granting institutions and 29 disciplines, we investigate which aspects of the academic workplace are (i) most gendered and (ii) most relevant to faculty retention patterns among respondents. Analyzing 6,615 free-text responses reveals that feeling devalued at work, both in formal evaluations and informal interactions, is the topic where women and men’s reported experiences diverge the most when given the opportunity to discuss anything that needed to be different about their jobs—a pattern that holds for nearly 300 institutions and across both STEM and non-STEM fields, and was particularly strong for women of color and tenured women in our sample. This is in contrast to most existing gender-based interventions in academia that target work–life balance. Further, devaluation is the topic most strongly associated with gendered departures among respondents, accounting for the majority of the gender gap in both considering leaving (current faculty) and leaving (former faculty). Finally, illustrative quotations contextualize gender-based devaluation in academia. These results provide new evidence for the long-known gender gap in faculty retention, illuminating how gendered dynamics in the workplace cross disciplines and institutions and relate to gendered decisions to leave academia.

Significance statement Women professors are more likely to leave their jobs than men. While many efforts to improve the retention of women faculty focus on work–life balance, our large-scale survey of current and former faculty across nearly 300 institutions reveals that the most gendered workplace factor among respondents is feeling devalued, both in formal evaluations and daily interactions. This pattern is strongest for women of color and tenured women and is surprisingly consistent across disciplines. Further, we show that devaluation is the most gendered reason in decisions to leave faculty jobs. By highlighting the distinct role devaluation may have in gendered departures, our findings provide actionable insights for policymakers and institutions hoping to improve equity in academic retention.

## Introduction

Women remain underrepresented among tenure-track and tenured faculty in nearly all academic fields, in part because they leave their faculty positions at higher rates than men (1–4). Despite decades of research, progress to mitigate gendered attrition among faculty has been limited because the experiences that lead to gender-based departures are numerous, multifaceted, and interconnected (5–15). As a result, we lack a systematic understanding of how gendered experiences in the academic workplace connect with decisions to leave across disciplines and institutions. In light of this complexity, there is a growing need to conduct broad studies of gender differences in experiences in academia that go beyond single-discipline and single-institution studies of gender gaps (16, 17).

However, large-scale quantitative studies that document patterns of gender disparities at particular career junctures, e.g. attrition, promotion, funding decisions (4, 18–28) often cannot tap into the underlying experiences that produce these disparities as they are limited to predetermined categories. On the other hand, small-*N* surveys and qualitative studies provide rich and open-ended details about women faculty’s workplace experiences, but only within a limited range of departments or disciplines (29–33), and often lack the statistical power to compare across groups. Furthermore, both qualitative and quantitative studies have tended to focus on early-career faculty, especially those in STEM disciplines and at prestigious institutions, which represent a minority of all faculty.

A deeper understanding of how gendered perceptions of the workplace intersect with faculty retention across a broad range of career stages, fields and institutions would guide policymakers and administrators in designing and testing meaningful, targeted policies to better retain women faculty. Such insights would also inform our scientific understanding of gendered workplace dynamics in the broader US workforce (34–38).

Here, we systematically investigate the ways that faculty respondents experience the academic workplace, and why they report leaving. We use detailed survey data on the experiences of a sample of current and former tenure-track and tenured faculty in the US, employed at nearly all PhD-granting institutions in 29 disciplines. These data include 6,615 free-text responses from the same participants when asked what needed to be different about their positions.

Our study combines the breadth of a large-scale, multidisciplinary study with the explanatory depth of qualitative research, enabling us to rigorously examine how and why the experiences of women faculty in our sample may differ from men’s across a broad range of disciplines, institutions, and career stages. With this unique dataset, we use a bottom-up approach to inductively analyze the free-text responses and develop an expansive understanding of the aspects of the workplace faculty respondents find most relevant to their retention, based on respondents’ self-reported experience and identities.

## Reasons for leaving: themes and topics

### Survey design

To elucidate the reasons faculty leave their positions, we conducted a large survey during the fall of 2021 on a frame of all current and former tenured and tenure-track faculty across 29 academic fields, representing eight broad domains of study, at all 391 PhD-granting institutions in the US (Section S1A). Fields were chosen to represent a diverse range of sizes, norms, and scholarship. Respondents reported their self-identified gender, race, and parenthood status, which we used in our analyses. Our study design allowed us to differentiate between current faculty who did not leave their position between 2011 and 2021, faculty who switched institutions within the set of US PhD-granting institutions, those who left academia, and those who retired (Fig. S1). Current faculty also reported whether they intended to leave their jobs.

Former faculty were identified from employment records as individuals who appeared in the survey frame in prior years but were no longer affiliated with a US PhD-granting institution at the time of the survey; this included those who had left academia entirely, moved to non-PhD-granting institutions, or retired (see Section S1A or Ref. (39) for details).

We note that the population studied here—tenured and tenure-track faculty at PhD-granting institutions—represents a small share of the academic workforce, excluding teaching track faculty, contingent and soft-money faculty, and adjuncts. Our findings should therefore be interpreted as specific to this population of respondents rather than as representative of the academic workforce more broadly.

The frame we sampled from was collected by Academic Analytics Research Center, which we subsequently cleaned, preprocessed, and validated (Section S1A). For each faculty member in one of the 29 selected fields, we utilized a combination of web scraping and crowd workers to retrieve their email address (Section S1B). A significant share of faculty do not have publicly available email addresses, which varies widely by field (Table S1).

We evaluated the observable differences between the larger population (Section S1A), the survey frame (Section S1B), the survey respondents, and the free-text respondents along four key variables: gender, academic rank, STEM vs. non-STEM, and a measure of institutional prestige derived from field-specific faculty hiring networks (18) (Table S5). The nonuniform availability of emails makes the survey frame slightly different in some ways from the census population, in that (i) full professors were somewhat overrepresented, (ii) assistant professors were somewhat underrepresented, and (iii) professors from higher-prestige institutions were overrepresented (Table S5). Otherwise, our analysis suggests that the survey frame was broadly representative of the census population. In our analyses, we examine differences across career age and institutional prestige to mitigate unbalanced representation across these variables.

The survey response rate was 14.1%, consistent with other large-scale studies of faculty and scientists (40, 41). Table 1 summarizes the degree of selection at each stage of the study, from the census population of faculty to the subset who provided free-text responses. While survey respondents were broadly comparable to the frame on several observable characteristics (Table S5), unobservable differences may remain, including, for instance, the possibility that respondents who left free-text answers differed systematically from those who did not, or that respondents were less likely to emphasize work–life balance concerns than nonrespondents. Results should therefore be interpreted as providing deeper insight into the experiences of a subset of faculty rather than as causal explanations of gendered faculty departures. We also note that this survey was conducted during the COVID-19 pandemic, which may have shaped how respondents described certain workplace experiences.

**Table 1:** Selection stages from census population to analytic sample.

Stage	*N*	% of prior stage	% of population	% of survey frame
Census population	145,455	–	–	–
Survey frame	71,205	49	49	–
Survey respondents	10,071	14.1	6.9	14.1
Free-text respondents	6,615	65.7	4.5	9.3

Each row shows the number of individuals at each stage of sampling and the share sampled relative to the prior stage.

Even so, the scale of the data is sufficiently large and the responses sufficiently detailed that analyzing it provides valuable new insights into the reasons for faculty departures.

### Survey respondents

This study focuses primarily on the free-text responses to an open-ended survey question, which asked current faculty what needed to be different to reduce negative experiences in their positions (N=4,442). Their responses are complemented by those of faculty who switched institutions, left academia, or retired, who were asked what needed to be different to have stayed in their positions (N=2,173).

Survey respondents represented a broad range of career stages, fields, and institutions, which allowed us to conduct detailed comparative analyses. Respondents ranged in career age from 1 to 40+ years after receiving their PhD. White men respondents were, on average, older than white women, men of color, and women of color (Fig. 1A; top left). The majority of respondents were current faculty at the time (66% of respondents). Among current faculty respondents, men comprised 60%, women 40%, and nonbinary faculty <1%; among former faculty (34% of respondents), men comprised 65% and women 35% (Fig. 1A; top right). Representation of respondents by race and gender varied widely by domain of study. Math & computing, engineering, and the natural sciences had the largest share of respondents who identified as white men and education had the smallest (Fig. 1A; bottom right). Respondents also worked at 311 unique US PhD-granting institutions. We derived a unique prestige score for each institution from field-specific faculty hiring networks (18). Survey respondents were much more likely to work at higher-prestige institutions than lower-prestige institutions (Fig. 1A; bottom left).

**Figure 1:**
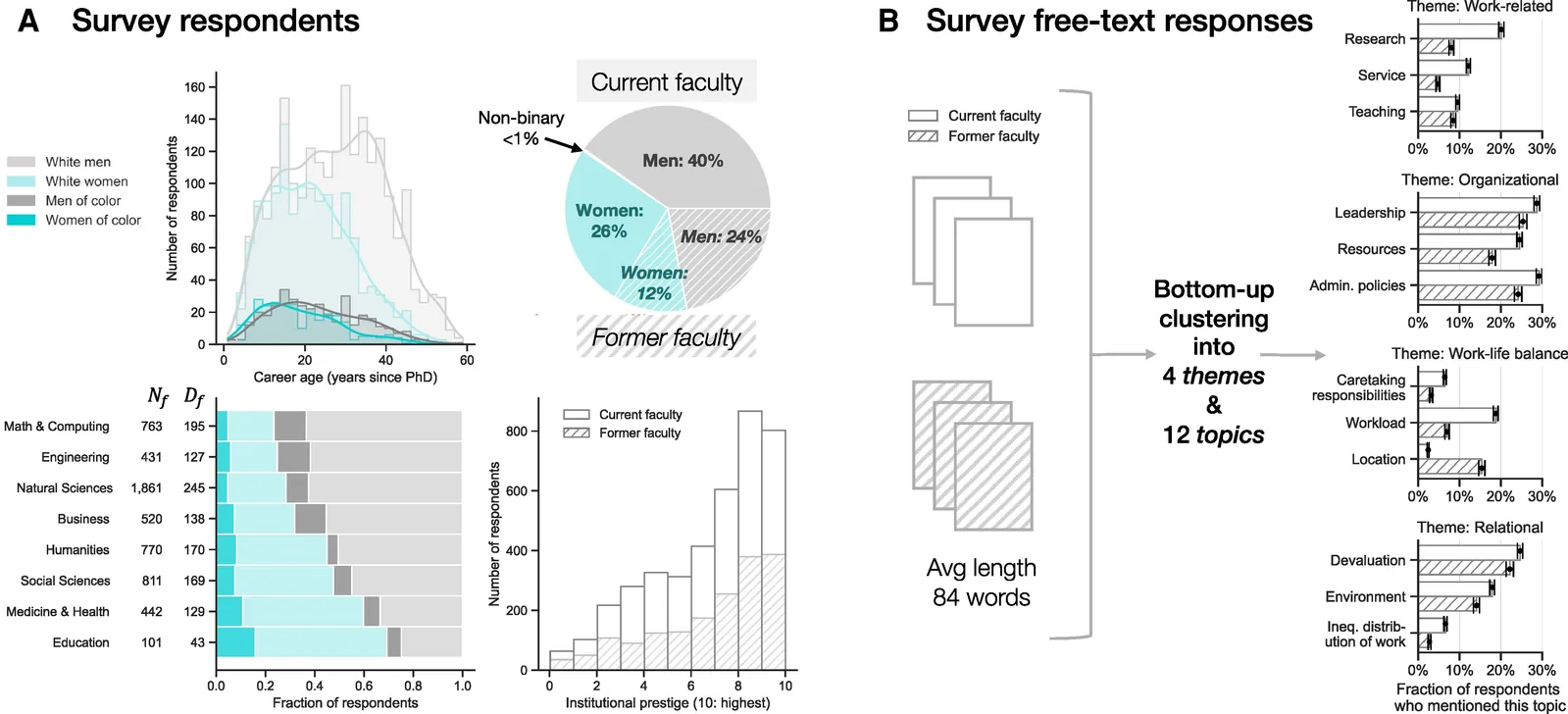
Survey respondents and responses. A) Overview of survey respondents who answered the free-response question (N=6,615): (top left) Number of respondents by self-reported gender and race across career age, defined as years since receiving their PhD; (top right) Share of respondents by self-reported gender and whether they were current or former faculty at the time of the survey; (bottom left) Share of respondents by self-reported gender and race across domains of study. $N_{f}$ represents the number of unique faculty respondents within that domain, and $D_{f}$ represents the number of unique departments within each domain; (bottom right) Number of current and former faculty respondents across institutional prestige decile, with 0 representing the lowest and 10 highest prestige. B) For each response (N=6,615; average word count 84 words), two co-authors independently read and categorized the response into four high-level themes (work-related, organizational, work–life balance, and relational) and 12 topics within those themes. Experiences could be categorized into multiple topics (i.e. these themes and topics are not mutually exclusive). Additional topics surfaced specific to current vs. former faculty; only overlapping topics are shown here. Each topic contains several subtopics. Coding disagreements were resolved via group discussions. See Methods for more details on the study design, codebook construction, and data analysis process, and Section S2A for the codebook and definitions (Table S7).

### Clustering data into themes and topics

To perform our analysis of the free-text responses, we inductively identified themes from the responses using a bottom-up approach (42). An important benefit of this approach is its ability to extract nuance from responses in a way that sheds light on social processes and is not limited to themes only in prior work.

Faculty respondents described a wide range of experiences in their free-text responses (Fig. 1B). *Work-related* topics included research, such as funding, publishing, and running a lab, as well as service and teaching. *Organizational* topics included concerns with departmental or institutional leadership, resources such as salaries, administrative support or facilities, and issues with specific administrative policies, such as sabbaticals, parental leave, and evaluation metrics. *Work–life balance* topics included caretaking responsibilities, workload (amount of work), and geographic location (e.g. living in a rural area or far from family). Finally, *relational* topics that involved social interactions and relationships at work included individual feelings of devaluation, generalized concerns with the departmental environment, and the inequitable distribution of work among colleagues.

Two co-authors independently read and categorized each response (N=6,615) into these four high-level themes (work-related, organizational, work–life balance, and relational) and 12 topics within those themes. Experiences could be categorized into multiple topics (i.e. these themes and topics are not mutually exclusive). Each topic contains several subtopics. See Methods for more details on the study design, codebook construction, and data analysis process, and Section S2A for the codebook and definitions (Table S7).

### Response differences

Current and former faculty in our sample were equally likely to mention organizational topics like concerns with their institutional leadership and resources, teaching pressures, and relational topics like devaluation and the departmental environment (Fig. 1B). However, current faculty respondents were more likely than former faculty respondents to mention research pressures, service responsibilities, caretaking responsibilities, their workload, and the inequitable distribution of work. Former faculty respondents were more likely than current faculty respondents to mention administrative policies and their geographic location.

Most topics were consistently reported across disciplines, with the exception of research pressures, such as obtaining research funding, which was mentioned more often by faculty in STEM disciplines (Figs. S7 and S14). In addition, several topics varied across institutional prestige, with issues with leadership and resources more frequently reported by faculty in our sample working at lower-prestige institutions (Figs. S9 and S16).

## Gendered experiences in academia

The topics respondents discussed are well-documented in the literature. Further, many of these topics are known to be gendered and racialized. Caregiving responsibilities in particular tend to fall disproportionately on women (21, 31), which was especially the case during the height of the COVID-19 pandemic (32, 43–45), when our survey was administered. Service and teaching responsibilities are heavier for women, especially women of color (19, 20, 29, 30, 46). Women can be excluded from academic social networks, and can regularly experience subtle exclusionary behaviors or microaggressions, which can create a so-called “chilly climate” for women faculty, especially women of color, and can lead them to consider leaving their jobs (33, 47–50). Despite an established literature on these topics, a comparative analysis is needed to identify which of these experiences are most gendered, and which are most relevant to faculty retention.

In our sample, the categories with the largest gender differences (the percentage of women who reported the topic minus the percentage of men who reported the topic) are devaluation, administrative policies, the inequitable distribution of work, service, and caretaking responsibilities (Fig. 2A). The only topic that men in our sample discussed more than women was research pressures, which was driven by early- and mid-career men (Fig. 3A). This topic is notable in that it is gendered, but in the opposite direction as all other gendered topics. Several topics were only marginally gendered, such as teaching and geographic location (Fig. 2A). Concerns with institutional leadership was the least gendered topic in our sample, reported in nearly equal frequencies over career age (Fig. 3C) and the only topic without a statistically significant gender difference in frequency (Fig. 2A).

**Figure 2:**
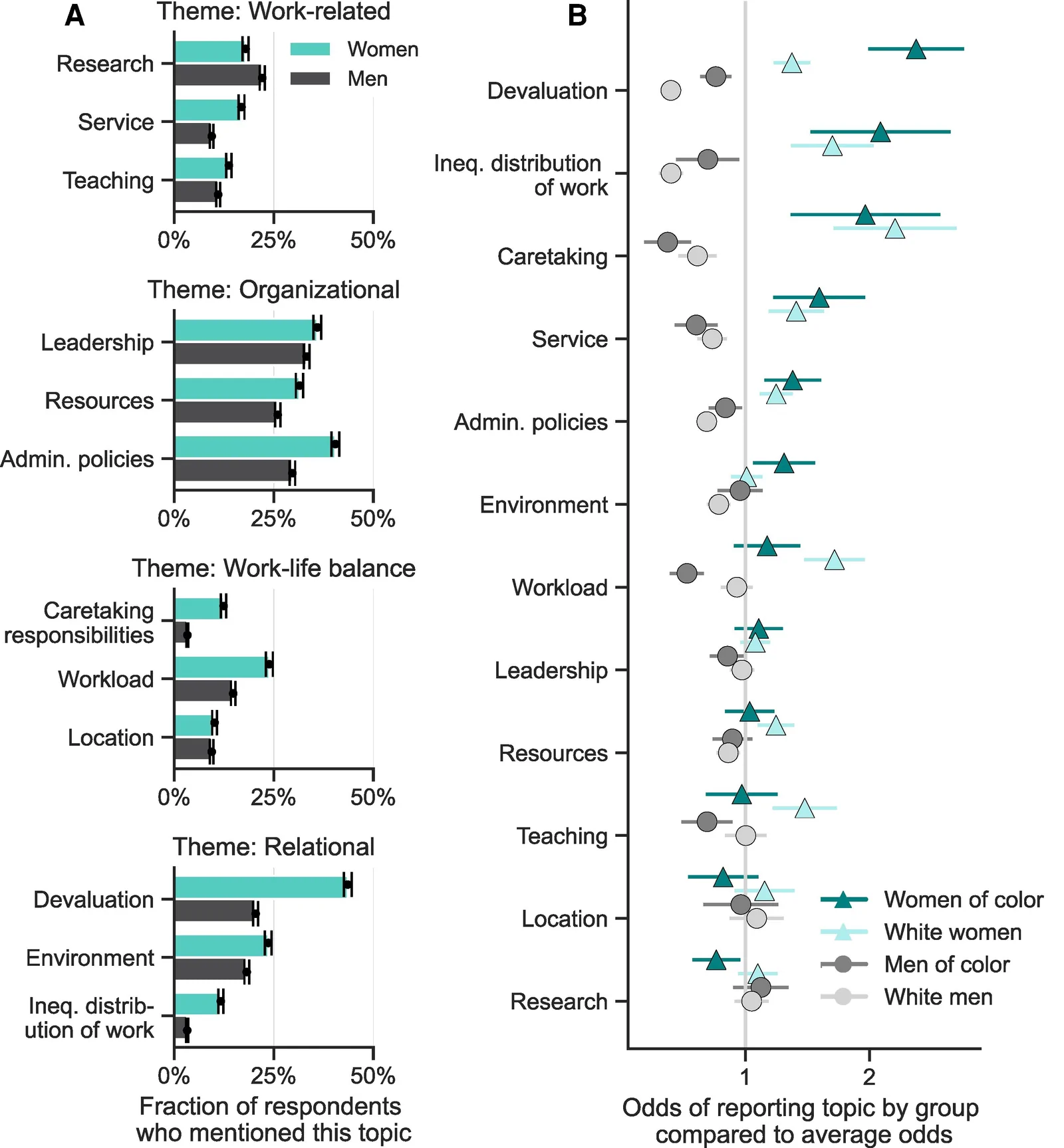
Group differences across topics. A) Frequency counts of topics described by faculty (N=6,447; 168 responses excluded, see Section S1B), split by self-reported binary gender. All gender differences are statistically significant at α=0.05 via two-proportion *z* tests with the exception of leadership. 95% CI shown. B) Odds of each group of faculty (women of color, white women, men of color, and white men) discussing each topic, divided by the mean odds for all faculty, adjusting via logistic regression for career age, discipline (one of 29), unique institutional prestige scores for each institution, and whether they are current vs. former faculty (Section 3A; Table S13). 95% CI shown. An odds ratio of 1 means that group was no more likely to mention the topic than the average faculty member. Note: current and former faculty grouped together; see Section S4, Figs. S2 and S10 for disaggregated results.

**Figure 3:**
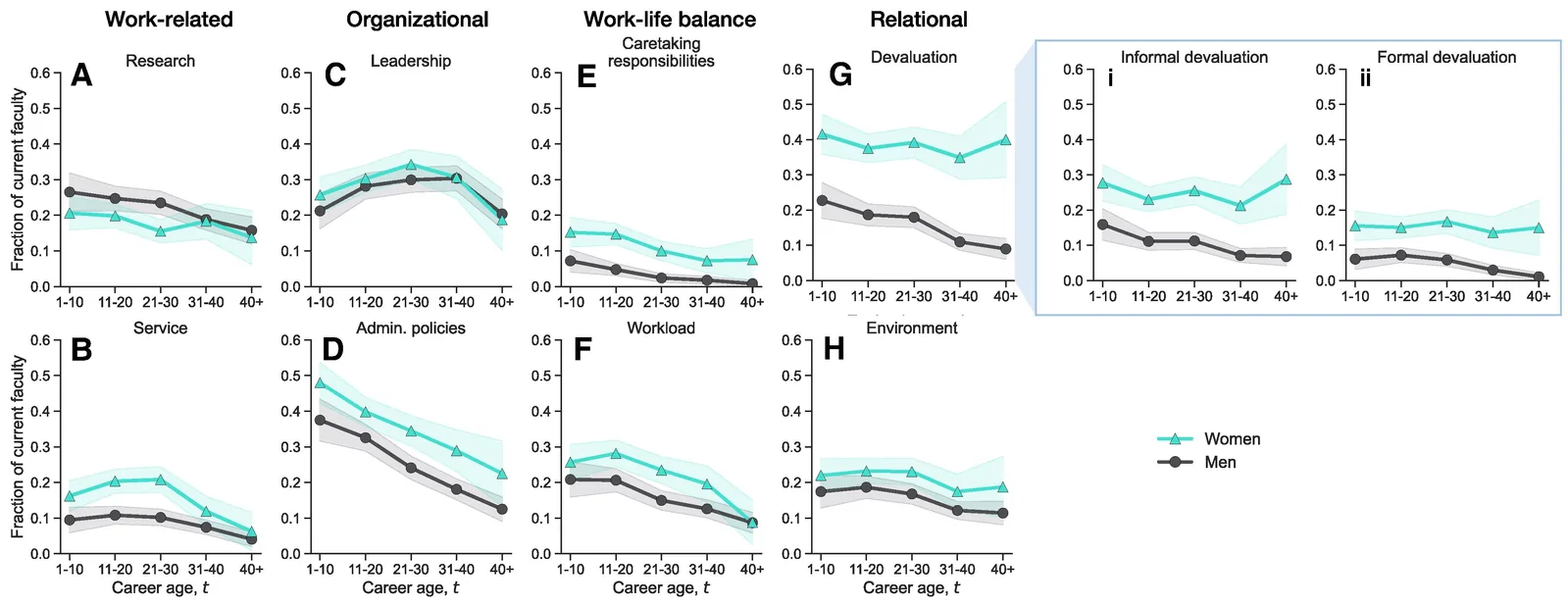
Topic differences by seniority. Fraction of current faculty (N=4,274), split by gender, who reported selected topics (see all topics in Fig. S6), across career age, *t* (years since receiving their PhD), binned into decades, and grouped by work-related topics (A, B), organizational topics (C, D), work–life balance topics (E, F) and relational topics (G, H). Inset shows devaluation split by (i) informal devaluation vs. (ii) formal devaluation. Envelopes indicate 95% CI. Former faculty in Fig. S13.

Differences in topics may be explained by variation in other factors, such as faculty age, race, field, or institution, and only incidentally correlate with gender. Using logistic regression with adjustments for faculty career age, discipline, institutional prestige, and whether they are current or former faculty, we computed the relative odds of reporting each topic per group of faculty respondents (women of color, white women, men of color, and white men) compared to the average respondent (Fig. 2B).

We find that even after adjusting for these factors, the most gendered categories are still devaluation, administrative policies, the inequitable distribution of work, service, and caretaking responsibilities. However, interesting patterns emerge around race. For instance, the odds of white women reporting devaluation decrease after adjusting for these covariates, and the odds of women of color reporting devaluation increase. The topic with the starkest contrast between white men and white women is caretaking responsibilities, while between white men and women of color it is devaluation (Fig. 2B).

It is notable that the topic of caretaking responsibilities is consistently gendered over career age (Fig. 3E), but is mentioned much less often than nearly every other topic, a surprising finding given the focus on work–life balance interventions for gendered faculty retention. In contrast, the topic of administrative policies is consistently gendered over career age (Fig. 3D) and is also the highest of all the topics in early career, even higher than devaluation (Fig. 3D and G).

Although we find other gendered aspects of academia, particularly the inequitable distribution of work and caretaking responsibilities, we focus subsequent analyses on devaluation in particular because: (i) it is the aspect overall we find has the largest gender difference (see Section S2B for additional calculations of difference), (ii) it is the least explored topic in the literature, whereas the gendered impacts of inequitable workloads, service, and caretaking are all well-documented, and (iii) it is the topic most often discussed by both current women faculty considering leaving their jobs and former women faculty who left their jobs in our sample.

### Devaluation

Out of all topics discussed, we find that devaluation—defined as the experience of feeling excluded, disrespected, mistreated, or held to different standards—was the most distinctly gendered topic in our sample (38% of women vs. 16% of men reported experiencing devaluation; z=16.9, N=4,274, P<0.001; Fig. 2A; nonbinary faculty excluded due to sample sizes, Fig. S3). In fact, women’s odds of reporting devaluation were 3.4 times higher than men’s (z=18.2, N=3,992, P<0.001; Section S3B, Table S14) in a logistic regression analysis adjusting for a number of covariates.

While faculty respondents of all backgrounds reported feeling devalued for a variety of reasons, often unrelated to their identities, the reports of women were distinct, in that their perceived experiences of devaluation were often explicitly tied to their gender, race, age, or parenthood status (52% of women vs. 13% of men who reported devaluation reported it was identity-based; z=12.6, N=1,041, P<0.001; Section S2B).

Devaluation is also the most racialized aspect of the academic workplace, with nearly half of women of color in our sample reporting devaluation (48%), making them the group most likely to report devaluation, followed by white women (36%), men of color (24%), and white men (14%). This pattern varies by race, with 76% of Indigenous women reporting devaluation, compared to Hispanic women (51%), Black women (51%), and Asian women (41%; Fig. S4).

In contrast, mothers and fathers in our sample report devaluation at similar rates as nonmothers and nonfathers (fathers slightly more than nonfathers; Fig. S5). However, mothers report issues with administrative policies (e.g. parental leave), and managing their caretaking responsibilities and workloads more than all other groups of faculty (Fig. S5).

Surprisingly, devaluation was consistently higher for women respondents compared to men in both STEM and non-STEM fields (Figs. 4 and S7). Even so, some fields have larger gender gaps than others—the differences in rates of devaluation reports among current faculty between women and men were 39% in engineering and 36% in economics, compared to just 15% in psychology and 18% in computer science (Fig. S8). This pattern is uncorrelated with the fraction of women in a field (R=−0.3,P=0.2), evidence against purely compositional explanations of devaluation such as tokenism. Similarly, we found a large and prestige-independent gendered difference in reported devaluation among respondents (Figs. S9 and S16).

**Figure 4:**
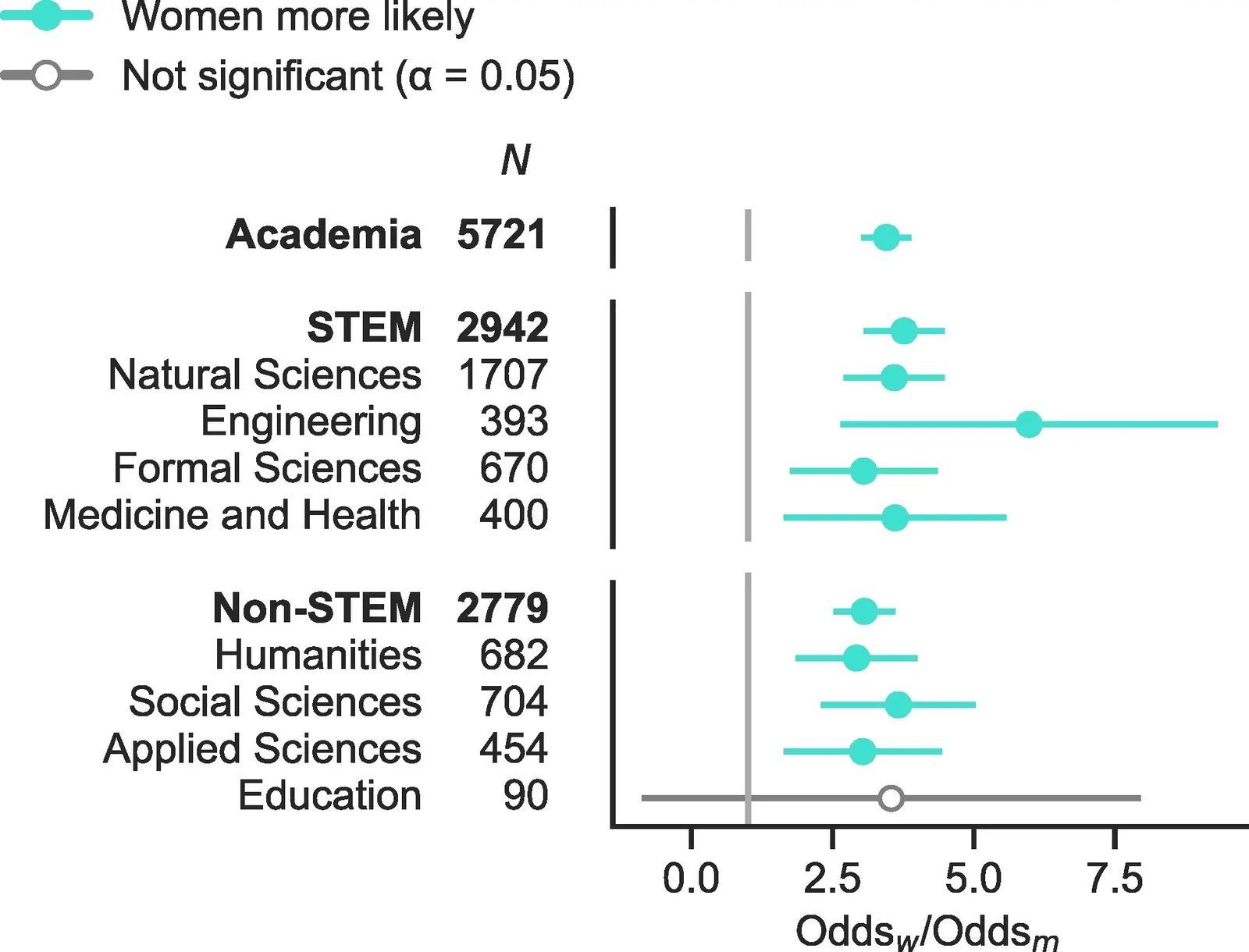
Gendered odds of reporting devaluation across disciplines. Odds ratios for reporting devaluation in each discipline (Section 3B; Tables S15 and S16), adjusting via logistic regression for gender, race, career age, discipline, institutional prestige, and whether they were current vs. former faculty, with 95% CI, and statistical significance assessed via a z test.

Women respondents report devaluation consistently across career age, while reports of all other topics generally decrease with age (Figs. 3 and S6). In fact, the gap in reported devaluation between men and women grows with career age—for respondents in the first decade of their careers, the gender difference in reported devaluation is just 19% but grows to 31% for respondents at the end of their careers (Fig. 3G), a pattern no other topic exhibits (Fig. S6).

Devaluation can be subdivided into formal and informal experiences. Interventions tend to focus on addressing formal devaluation, such as changing formal evaluation processes (7, 51, 52). However, we find that women in our sample across career stages report informal devaluation, such as microaggressions, more often than formal devaluation (Fig. 3 inset), a pattern that holds across subgroups (Figs. S10 and S18). The qualitative accounts in the next section, and Tables S8 and S9 illustrate both forms, with women describing formal devaluation through biased evaluations and informal devaluation through social exclusion.

Devaluation is related to epistemic exclusion, an existing theoretical framework that characterizes the way the scholarship produced by faculty of color is devalued through the intersection of formal institutional evaluative structures and individual biases (53, 54).

We group formal and informal experiences of devaluation into one conceptual category because the boundaries between experiences which met the legal definition of harassment or discrimination vs. subtle exclusionary behaviors are often blurry. Instead of creating an artificial distinction between them, we combined them into one category, where they are considered equally regardless of intensity, consistent with recent work demonstrating that mistreatment is not cut and dry (55, 56).

### The connection between devaluation and gendered departures

Among both current and former faculty in our sample, we observe gendered retention patterns—women are more likely than men to leave and consider leaving. Current women faculty respondents who mentioned leaving their jobs were more likely to discuss devaluation (56% vs. 36%; z=5.0, N=643, P<0.001) and leadership (46% vs. 28%; z=4.9, N=503, P<0.001) than women who did not mention leaving. In contrast, among men in our sample, no topics were disproportionately discussed by those who mentioned leaving compared to those who did not.

Among current faculty respondents who considered leaving, what topics were they most likely to discuss? We computed conditional probabilities P(T| mentions leaving) for each topic *T*, split by gender (Fig. 5A; Section S3C). Women in our sample who considered leaving were most likely to mention devaluation (particularly informal), and leadership. Men who considered leaving were most likely to mention leadership, followed by devaluation.

**Figure 5:**
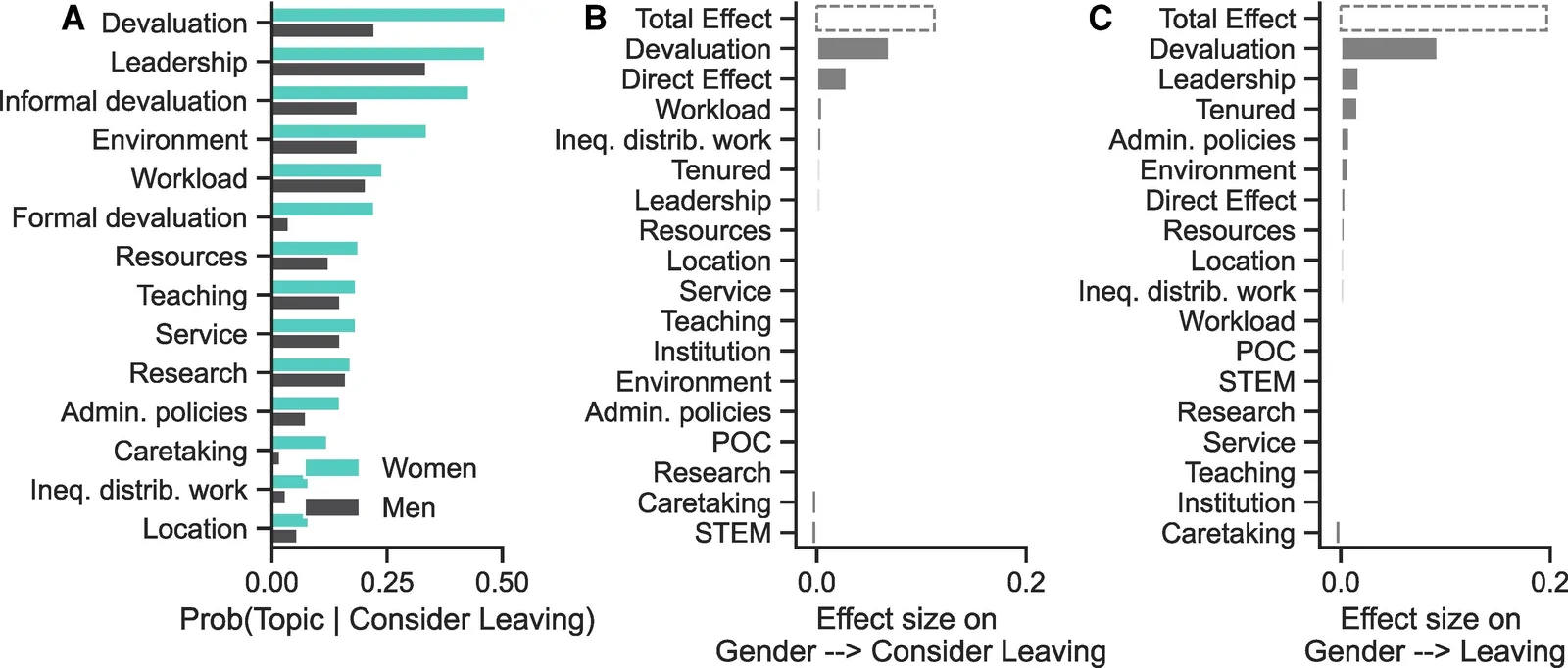
Devaluation is the strongest factor in gendered retention patterns in our sample. A) Conditional probabilities of discussing a topic among current faculty respondents who indicated they were considering leaving their jobs, by binary gender. B) Total and mediated effect sizes among current faculty for associations between gender and considering leaving. C) Total and mediated effect sizes among former faculty for associations between gender and leaving. Regressions in mediation analyses control for all 12 topics in Fig. 1, race, discipline, institutional prestige, and career age (see Methods).

While these probabilities were calculated independently, a multiple mediation model allows us to examine which topics best explain the relationship between gender and retention while accounting for their potential interrelationships as well as other potentially influential covariates (57) (though we caution that in this case, these models are descriptive and should not be interpreted causally (58); see Limitations). We fit two separate models for current and former faculty. In each of the models, the retention outcome variable was defined differently. The model for current faculty estimated the effect of gender on considering leaving a position, while the model for former faculty estimated the effect of gender on actually leaving a position. We included all 12 topics as well as faculty race, discipline, institutional prestige, and career age in the models. See more details in Section S3C (Tables S17 and S18).

We find that, in our sample, devaluation has the largest indirect effect on the relationship between both gender and considering leaving (current faculty) and gender and actually leaving (former faculty), supporting its role as the dominant explanatory factor in the models. In both models, the association between devaluation and gendered retention was larger than the combined associations of the other 11 factors, including highly gendered experiences such as the inequitable distribution of work and caretaking responsibilities (Fig. 5). The indirect effect of devaluation also exceeded the direct effect of gender on retention, suggesting that observed workplace experiences—particularly devaluation—account for more of the gender gap in retention than unobserved factors in our sample.

***Did faculty who considered leaving actually leave?*** Our survey responses were retrospective. It is possible that looking back, feeling devalued weighed more heavily in respondents’ reflections on their decisions to leave or consider leaving. Using employment records from 2021 to 2023 covering 97% of current faculty respondents, we identified whether they left their jobs in the 2 years following the survey. We found that among current faculty respondents in 2021 (N=4,222), 67 left their jobs between 2021 and 2022 and 46 between 2022 and 2023.

In the survey they took before they left, women who left (N=42) were most likely to mention devaluation (45%), whereas men who left (N=97) were most likely to mention leadership (34%), and only 14% mentioned devaluation. The gendered difference in reporting devaluation was significant via a 2-proportion *z*-test (z=4.1,P<0.001).

Because attrition is relatively rare over this 2-year window, we lack the statistical power to draw strong conclusions from these data alone. Nonetheless, the statistically significant gendered difference in proportions offers some reassurance in the validity of our mediation analysis of former faculty survey respondents (Fig. 5), as respondents’ retrospective reflections were likely representative of their feelings at the time of leaving.

## Contextualizing devaluation and retention

Here, we record the specific ways women and nonbinary faculty respondents across a broad range of career stages, fields, and institutions, reported experiencing gender-based formal and informal devaluation in academic workplaces, and what they suggest could be done to improve their experiences. They report that devaluation is exacerbated when leadership fails to respond to reports of devaluation and this often leads them to consider leaving their jobs. This section focuses primarily on current faculty, as they are the group *at risk* of leaving. We provide illustrative quotations in the main text and additional examples in Section S2C (Tables S8–S12). We include demographic identifiers for each quotation but may omit details to preserve the respondent’s anonymity.

### Formal devaluation

Women reported feeling like they need to meet higher expectations, with fewer resources and for fewer rewards, than their male colleagues. Women often feel that they need to be excellent to succeed, while reporting that men can be mediocre and still succeed (Table S8A). As one white tenured woman biology professor described:

I was told that I needed 15 papers to get tenure, when the 2 guys ahead of me got tenure with 2–3 papers [each]. I was told I needed this many papers to prove I could be productive “even with having a baby”…This is a constant refrain—standards in our department are only high for the women and men seem to be able to just trip over a bar that is at ground level and be highly rewarded and compensated.

Even though the expectations often feel higher, women also report feeling the need to meet these expectations with fewer rewards and resources than their male colleagues (Table S8B). For example, a Black tenured woman professor in the social sciences reported that:

I have to justify each year why a course release is required [for me] to properly run the program, yet [the white male director of another program] was freely offered what I had to fight to keep.

When they achieve despite these barriers, they report that their research achievements often go unnoticed or are devalued (Table S8C), for example, as one white tenured woman engineering professor said, “No matter how hard you worked and how great your proposal is, you received the CAREER award because you were a woman or minority.” And, in addition to research, women, especially women of color, often perform large amounts of “office housework” (59) or service that is not rewarded formally, but can negatively impact their evaluations if they say no (Table S8D). As a white tenured woman in chemistry summarized, “Men can generally be bad departmental citizens as long as their research is valued, but women can’t.”

Women also reported that workplace evaluations typically do not acknowledge the higher domestic workloads women, especially mothers, carry (Table S8E). Combined with the inequitable service load, one white tenured woman in sociology reported that “Many many women I know are simply burned out because they are often primary caregivers in their personal lives and are also caretaking their departments.”

These aspects combine to make women feel like the formal faculty evaluation processes are fundamentally unfair. Tenured women report that after successfully advancing through all of these barriers, they felt like their work was less worthwhile to the organization because of their gender. As a Hispanic tenured woman in chemistry reported:

In my department, meritocracy is the excuse for everything. But meritocracy is really just a way of supporting the status quo of a dominant white, male environment…I thought that if I worked hard enough, my work would be recognized. But, sadly, that is not how the system works. Looking back, it is clear from day 1 that the system wasn’t just not in my favor but was actively working against my success.

### Informal devaluation

In addition to feeling like their work is devalued, women often report not feeling valued as people, through both subtle exclusionary behaviors (i.e. microaggressions) in their daily interactions, as well as overt mistreatment. Women reported that subtle exclusionary behaviors are frequent, tiring, and accumulate over time (Table S9A). As a white pretenure woman in chemistry described:

When I started my position, I was one of 2 females in the department! I have gotten ALL the things: Left off invites to visit with out-of-town speakers, left off reception invites, comments ignored in meetings, left off important committees, explicitly told “I didn’t know [your university] hired female professors” and “I didn’t know you were a “real” professor”…Literally ALL the things. It is very tiring, to say the least.

Exclusionary behaviors were especially salient for mothers, women of color, and older women and did not always originate from men (Table S9B). As one Black tenured mother in the social sciences reported, gender-based exclusionary behaviors were sometimes perpetrated by women in leadership positions:

When I was starting out on the tenure track, family leave was discouraged in my department. I was told directly by my female chair and female senior colleagues that it would “look bad” if I took my legal right to family leave. It was one of the worst professional decisions that I ever made and it was absolutely brutal taking care of an infant while trying to get tenure.

In addition to subtle acts of devaluation, some women reported overt acts of gender-based harassment, mistreatment, and violence (Table S9C). As a white tenured woman in English literature described:

Some of my male colleagues harassed me, ignored me, derided my work or made the department a hellish place to work and teach, as I knew they were also harassing or even sexually abusing students, but I could never get any of them convicted. I survived, but not without rage and scars.

In summary, women faculty reported experiencing a range of gendered factors in the academic workplace that often combine to make them feel like their work and themselves as people are not valued, while their male colleagues do not report these factors nearly as often. When asked what needs to be different to reduce the stress they feel in their positions, one white tenured woman in mathematics exclaimed, “Climate!!! I am tired of feeling the need to justify my existence and prove my credentials on a regular basis.”

### Lack of institutional response to devaluation

Women report that the experience of devaluation—both formal and informal—is particularly impactful. But a lack of institutional response when they reported events or asked administrators for specific steps to be taken to address the devaluation they experienced was felt to inflict another, secondary harm (i.e. secondary victimization (60–62) and institutional betrayal (63–65)). Both women and men reported feeling frustrated that their input was not sought or valued by administrators (Fig. 2A). However, for marginalized groups, the experience is distinct, because the departmental and institutional changes they report asking for (e.g. to specific policies or practices) are often intertwined with their social identities.

When women identified systemic issues (e.g. gendered salary gaps) and asked for steps to be taken to mitigate them, they often reported that their institutions respond with silence or dismiss their concerns (Table S10A). As one white tenured woman in physics described:

I have spent countless hours over many years supporting new faculty women being hired…because I know just how hard it is…[My] university has a strict equality [vs equity] policy, so if you even bring up the idea that women need help or are treated unfairly, it is immediately shot down. No one listens, no one cares…I love the students here and I love the work I do…but I am exhausted. I need the university to make us a priority.

Others reported that administrators’ commitments to fairness felt performative (Table S10B). As a Hispanic tenured woman in history explained:

My department has large disparities in promotion rates by gender and race. My presence as a [person of color] is often referred to as a sign of diversity, and yet my serious requests to review inequities in promotion have been ignored, denied, or seen as special treatment.

In addition to indifference to systemic issues, women also reported cases of administrators actively exacerbating harms (Table S10C). For example, reports of mistreatment were often not taken seriously. As one white woman professor in STEM described:

I do not feel supported here. The time when the university’s true colors were shown to me was after the workforce violence incident, when the graduate student who committed the violence was not expelled and allowed to remain in my midst, where I continued to be afraid of him. If he, that violent aggressor, was removed from my workplace, it would have made a huge difference.

The lack of action by administrators can also, in turn, increase gendered service burdens, as women take on more work to try to improve their workplace climates. As a tenured Asian woman in anthropology described, “I have taken on leadership roles to try…to fix the system (and attitudes) at my own institution. I often feel like that is not enough.”

Finally, feelings of devaluation are heightened when institutional structures do not keep women who report gender-based mistreatment safe from retaliation. As one white tenured woman in STEM shared:

A department chair discriminated, retaliated against, and harassed multiple women. These women came forward. The university found no wrongdoing. The women, on the other hand, were labeled as troublemakers, and false narratives were written about them in permanent university documents. Administrators backed up the chair. Women’s careers are meaningless and can be destroyed, while the institution claims to care about equity.

### Retention

Respondents across a broad range of fields and institutions described the implications of the combination of sustained systemic gendered devaluation over a career and the lack of institutional response. One of the most pressing implications is that women faculty often question whether they want to stay at their institutions or in academia, even if they have already obtained tenure (Table S11A). As one white tenured woman in social work described feeling formally devalued:

I’ve had to fight for every raise…I’ve received. It’s this kind of nonsense that has made me realize over time that the university does not have my back with respect to gender equity. If I could see leaders in the higher ed space trying to solve these problems in earnest and large-scale ways, I may be tempted to stay.

A white tenured woman in physics shared a similar sentiment as she described feeling devalued informally:

My stress would be reduced if there were not pervasive harassment and discrimination. I think that will happen when pigs fly…The problem is that the senior white men in my field are actively opposed to retaining women and people of color. Department, college, and university leadership think that placating the senior white men is the top priority. So I’m interviewing for other jobs, and considering leaving academia.

Nonbinary faculty express similar sentiments (Table S11A). However, reports of considering leaving due to devaluation and a lack of institutional response were particularly salient for women of color (Table S11B).

While the prior quotations have focused solely on current women faculty and their experiences at work, we find that former faculty (N=2,197) report leaving their jobs due to similar, and often more intense, experiences of devaluation and a lack of institutional response—nearly half of women who left academia reporting leaving due to devaluation (48% of women vs. 22% of men who left academia; z=5.1, N=346, P<0.001; Section S4B). These results show that women faculty who experience devaluation not only consider leaving their positions, but they often actually leave, either for a different institution, to retire early, or to leave academia entirely. Each member of the faculty who feels pushed to leave their job is an unnecessary loss of expertise, institutional knowledge, future scholarship, and mentorship, and likely represents a great cost to the institution. Addressing the causes of gendered faculty attrition is thus likely to benefit institutions in many ways.

## Discussion

These results provide new evidence for the long-known gender gap in faculty retention, illuminating how gendered dynamics in the workplace cross disciplines and institutions and relate to gendered decisions to leave academia.

Our large-scale qualitative analysis of respondents’ free-text shows that devaluation represents a distinctly gendered aspect of the academic workplace, even after controlling for discipline and institution (Fig. 2B). Although work–life balance is the most common explanation in the literature for women faculty’s higher attrition rates (6, 8, 66, 67), these results indicate that work–life balance concerns were more relevant for early-career white women, but for all other women in our sample—particularly women of color and tenured women, devaluation was more influential (Fig. 2B).

We show that feeling devalued, both formally (Table S8) and informally (Table S9), is the most gendered experience reported by our sample, and the only topic that grows more gendered with seniority (Fig. 3G), suggesting that the accumulation of these experiences may drive the increased rate of gendered attrition among late-career faculty (4).

Women faculty across a broad range of backgrounds and departments report devaluation, but not all women experience it with the same intensity. Nearly half of women of color report devaluation, compared to just one-third of white women, a pattern that is even stronger for Indigenous, Black, and Hispanic women (Figs. S4 and S11).

While prior studies and interventions tend to focus on women in STEM, we found that devaluation was consistently high for women in both STEM and non-STEM fields (Fig. 4). However, we do find substantial heterogeneity across fields—for example, among current faculty, gender differences in reported devaluation are largest in engineering (STEM) and economics (non-STEM), and smallest in computer science (STEM) and psychology (non-STEM; Fig. S8). This heterogeneity is not explained by the gender composition of a field: the size of the gender gap in devaluation is uncorrelated with the percentage of women in a field (R=−0.3,P=0.2). This is inconsistent with a purely compositional account of devaluation, e.g. that it is driven by tokenism or a scarcity of same-gender role models, and points instead toward field-level or department-level norms around inclusion, competition, and collaboration, which will likely require additional data to untangle.

These results provide specific insights into how gendered and racialized experiences in academia connect to faculty departures. A multiple mediation model (57)—applied here as a descriptive tool to characterize associations rather than to establish causal claims—suggests devaluation has a distinct role in gendered retention patterns. Out of all job concerns discussed by respondents, devaluation is the topic most strongly associated with gendered retention, both for current faculty considering leaving a job and former faculty who actually left. This association was larger than the combined associations of the other 11 topics, including highly gendered experiences such as the inequitable distribution of work and caretaking responsibilities (Fig. 5).

For many respondents, the adverse impact of devaluation is exacerbated when administrators and other faculty fail to respond to reports of systemic devaluation or mistreatment (Table S10) (i.e. secondary victimization (60–62) and institutional betrayal (63–65)). Women explicitly connect instances of formal and informal gendered devaluation and instances of requests for change being ignored with considering leaving their faculty jobs (Table S11). Related work on gendered dynamics in labor markets more broadly reveals many parallels with our findings about gendered devaluation in academia (34–38), suggesting that a lack of organizational response to reports of devaluation at work affects women’s decisions to leave their jobs across many types of occupations (60–65).

The most effective intervention to improve relational dynamics in academic workplaces and better retain women faculty may be a commitment by administrators to address devaluation via organizational change. Specifically, our respondents report that they need their universities to (i) enforce procedural fairness in formal evaluation processes, (ii) create collegial, inclusive environments free of subtle exclusionary behaviors, and (iii) substantially improve misconduct reporting structures (Table S12). Many relational aspects that are relevant to gendered attrition operate at the department level, and climates can vary across departments even at the same institution (68), an idea supported by our survey responses. Hence, administrators will likely need to facilitate organizational change at the department level. Deans, department chairs, and other administrative leaders are particularly well-positioned to act on these findings. Understanding how everyday leadership behaviors can foster climates in which devaluation goes unaddressed is a critical first step toward institutional change, particularly given the current pressures facing higher education. Our survey responses indicate that all faculty, including men, can experience devaluation. Improving procedural fairness, departmental environments, and misconduct reporting processes should provide benefits to all faculty.

Our results suggest that faculty and administrators who wish to improve relational dynamics in a department could prioritize responding to devaluation in both formal and informal contexts, via concrete, systematic processes, and they should avoid performative or ad hoc approaches (Tables S10–S12). Most interventions focus on individual-based approaches in which women faculty often expend additional labor in order to receive the benefits (e.g. workshops, training, and individual conversations). Women in our sample report that interventions may be more effective if these traditional interventions aimed at individuals are coupled with “top-down” organizational policies and changes in management (69, 70).

Our findings are complemented by a growing literature on what to do about devaluation, both formal and informal. To increase fairness in formal evaluations, this literature currently recommends: (i) ensuring that consistent criteria are stated and applied in formal evaluations (51), (ii) removing thresholds related to bibliometric measures, e.g. h-indices, numbers of publications (51), (iii) adopting inclusive definitions of meritorious scholarship (51, 52), (iv) adopting gender-equitable norms around service and DEI work and how it is valued (51, 52), and (v) implementing standardized processes for pay raises regardless of external offers (7). Recommendations from the literature to mitigate informal devaluation include: (i) setting and maintaining norms to include women in academic social networks (52, 71), (ii) ensuring mentorship regardless of gender (52, 71), (iii) diversifying faculty hires (71), (iv) increasing awareness of gender bias among male faculty (72), and (v) consistently including all faculty voices in departmental and institutional decision-making processes (71).

Our results demonstrate that certain groups of faculty experiences in academia, such as many women’s experiences, particularly women of color, are distinct from other groups in ways that may not be visible to them. For academia to achieve the organizational change that women report is necessary to reduce the greater devaluation they report experiencing, faculty of all backgrounds need access to the particular gendered aspects of faculty life that they may not experience to the same extent in their own careers. Exemplar quotations were chosen in part to provide such a view, which is not possible to describe with quantitative evidence alone.

### Limitations

Our analyses included only tenured and tenure-track faculty at US PhD-granting institutions in 29 disciplines. Future research may consider investigating whether the topics identified here are similarly important for other populations, such as research or teaching-track faculty, or adjunct faculty. More broadly, our analytic sample represents a population that has already survived multiple selection stages in academia. Faculty who experienced devaluation early in their careers may have left before reaching tenure-track positions, meaning our estimates of the prevalence and impact of devaluation may be conservative.

As a group, our respondent population differed slightly from the census population of faculty we surveyed. Specifically, full professors were somewhat overrepresented, assistant professors were somewhat underrepresented, and professors from higher-prestige institutions were somewhat overrepresented (Table S5). To mitigate these representation differences, we split analyses by career age and institutional prestige or otherwise adjusted for these factors in our analyses. It is important to note that our definition of career age does not capture changes over a single person’s career but rather reflects cohort differences along with seniority differences.

As with any large-scale survey, potentially unobservable differences between our respondents and the full faculty population remain, which may have influenced our results. For example, if faculty parents were less likely to respond to the survey because of work–life balance issues, our respondent group may undersample faculty who emphasize work–life balance over relational dynamics. Likewise, faculty who chose to write free-text responses may have had stronger or more salient workplace experiences than those who did not, which could influence the distribution of topics we observe.

Our survey was also conducted during the COVID-19 pandemic, a period of unusual disruption to academic work. Pandemic conditions may have altered the salience of certain workplace experiences, particularly workload, caregiving responsibilities, and institutional support (43–45). Gender differences in pandemic-related caregiving burdens may have shaped how faculty described their experiences, though we note that devaluation emerged as the dominant concern even in this context, which may strengthen its relevance to gendered retention more generally.

Former faculty responses rely on retrospective accounts of why they left their positions, which introduces the possibility of recall bias (73–76). Nonetheless, our validation exercise using employment records from 2021 to 2023 provides some reassurance that retrospective accounts of former faculty were broadly consistent with contemporaneous reporting.

It is important to note that while devaluation was the most gendered topic, it was not reported by a majority of women in our sample. However, attrition is relatively rare, so focusing on the largest gender differences in experiences will likely inform our efforts to reduce gender differences in attrition.

Finally, mediation analyses have many limitations. We assume all relevant topics emerged from the data but that is likely not the case, so there could be unmeasured confounders (77). In addition, the interpretation of indirect effects in the model can be challenging, since we include 16 mediators (78, 79). A further concern is that simultaneously conditioning on multiple correlated mediators may introduce endogenous selection bias (58). For these reasons, the mediation results should be interpreted as descriptive associations that characterize which workplace experiences co-occur with gendered retention patterns in our sample, rather than as evidence of causal mediation. Future work studying the mechanisms of gendered faculty departures, involving longitudinal data and more sophisticated causal identification strategies, will be necessary.

### Conclusions

The topics faculty discussed in their free-text responses are generally consistent with those studied in prior work, even though we did not assume any themes existed in advance, confirming that many commonly studied aspects of the academic workplace are indeed gendered, such as caretaking responsibilities, service, and the inequitable distribution of work. We build on, extend, and add important nuance to those past findings (Figs. 2 and 3). However, our uniquely broad, numerous, and detailed responses also shed light on the distinctly gendered issue of devaluation, its consistency across disciplines and institutions, and its explicit connection to gendered faculty departures (Fig. 5), particularly for women of color and tenured women.

Our systematic investigation of professors’ experiences at work combines the breadth of large-scale analyses with the richness of qualitative methods. This combination represents a powerful approach for deeply understanding how gender dynamics vary across career ages, fields, and institutions. The results have widespread implications for our scientific understanding of gender and work, as well as specific implications for policymakers in higher education. We look forward to research that evaluates the efficacy of interventions to improve relational dynamics in academia and reduce experiences of formal and informal gendered devaluation at work.

## Materials and methods

### Survey design and analysis

Participants were identified from a census of all tenure-track and tenured faculty employed at all US PhD-granting institutions anytime between 2011 and 2020 (4, 18), obtained through a data-use agreement with Academic Analytics Research Center, which we substantially cleaned, processed, and annotated (Section S1A). To create the frame, we selected 29 fields across all eight domains of study. Fields were selected as a group prior to data collection to ensure diversity in size, gender composition, and disciplinary norms. In particular, fields were chosen to obtain (i) a mix of men vs. women-dominated fields, (ii) a mix of large and medium-sized fields, and (iii) a mix of commonly vs. uncommonly studied fields. We attempted to obtain email addresses for all faculty in those 29 fields using a combination of web scraping and an Amazon Mechanical Turk crowdsourcing task.

This resulted in a frame of 73,049 current and former faculty, who we emailed survey invitations from July to September 2021. Our final response rate was 14.1% (Section S1A; Table S2), similar to that of other large-scale surveys of faculty (40, 80), and on the upper end of the response rate range for surveys of authors of scientific publications. The response rate was higher for former faculty (those who left academia or retired; 34%) than the average (Table S3). The study protocol was approved by the University of Colorado IRB, protocol 21-0293, and informed consent was obtained from all participants.

### Qualitative design and analysis

Current faculty members answered the free response question, “What needs to be different in order to reduce the stress you feel in your position?” and former faculty members answered the related but slightly different free response question, “What needed to be different in order for you to stay in your position?” Both current and former faculty were informed their responses to the question would be used to develop solutions for faculty attrition. Not all survey respondents (N=10,071) provided qualitative responses (N=6,615; Table S5).

We analyzed responses to these questions separately for current vs. former faculty through three rounds of inductive qualitative coding (42). In the first round of coding, we began by developing codebooks of primary (i.e. top-level) and secondary codes for current and former faculty separately; each codebook was based on analysis of a test set of 100 responses and was refined through several rounds of testing and inter-rater reliability checks (Section S2A; Table S6). Two authors coded each response (N=6,615). The team met regularly to discuss code disagreements, and a mediator would decide when both raters did not agree (81). After completing this first full pass through all responses, we ran frequency analyses to determine which categories displayed the largest gender differences in reports. The secondary codes of service, informal climate, mistreatment, and formal evaluation practices were most gendered.

We then conducted a second round coding to capture themes in faculty experiences within these secondary codes. In this round, we followed the same procedure as in our first round of coding all responses: we developed a codebook of tertiary codes, also based on several rounds of testing on small test sets and inter-rater reliability checks (Table S7). This process resulted in several conceptual and structural changes to our initial codebook (see Section 2A).

We then hand-coded all text passages mentioning these secondary codes with their new applicable tertiary codes. After completing this second coding round, we ran frequency analyses of the codes, which showed devaluation was the code with the largest gender difference.

Finally, to capture themes and sources of variation within devaluation, we conducted a third and final coding round of quandary codes, following the same method, to capture the various ways that faculty discussed devaluation at work (Tables S8–S12).

In order to build trust with participants and to disclose our intended use of potentially sensitive information, when respondents were taking the survey the research team indicated that we would not share their quotations without seeking their explicit follow-up permission. Once we had selected an initial set of exemplar quotations, we emailed participants their quotations and asked for their permission. Most respondents gave us permission to use the entire quotation (82.5%), a few opted to edit their quotations to remove identifiable information (7.5%), and one respondent did not give us permission to use their quotation due to safety concerns (2.5%). The rest did not respond or emails bounced (7.5%).

When reporting quotations in our results, we default to including their gender, race (Asian, Black, Hispanic, or white), academic rank (pretenure or tenured), and field of study, including other identifiers such as parenthood status where relevant. However, during the quotation permission process they had the option to ask us to use a more coarse description of their identity when presenting their quotation (e.g. “woman of color” instead of their race, or “STEM”/“non-STEM” instead of their field). If a respondent’s quotation was particularly sensitive, we would use a more coarse description of their identity even if they did not request it.

### Quantitative analysis

All quantitative analyses were conducted in Python, using the statsmodels package for regression models and the semopy package (82) to conduct the mediation analyses.

#### Regression models

We were interested in comparing the frequency counts of topics reported by four groups: white women, women of color, white men, and men of color (Fig. 2B). In order to quantify which group of faculty was most likely to report a given topic *T*, we fit a separate logistic regression model to the dataset to model the probability *t* of reporting each topic *T*, adjusting for each group (white women, women of color, white men, and men of color; coded using sum-to-zero coding, see Section S3A), group type (whether they are current vs. former faculty), career age (years since PhD, ranging from 1 to 60), discipline (one of 29), and faculty employer prestige (scaled such that a 1 unit increase corresponds to a 1 decile increase up the prestige hierarchy, but note that each rank in the regression represents a unique institution; Section S1A):


$$\begin{array}{rl} \text{logit}(t) & =\beta_{0}+\beta_{1}[\text{Women of Color (WOC)}]\\ & \;+\beta_{2}[\text{White Women (WW)}]\\ & \;+\beta_{3}[\text{Men of Color (MOC)}]\\ & \;+\beta_{4}[\text{Group type}]+\beta_{5}[\text{Career age}]\\ & \;+\beta_{6}[\text{Prestige decile}]+\beta_{F}[\text{Discipline}], \end{array}$$

where *F* represents 29 different coefficients, one for each discipline. See Section S3A (Table S13) for more details.

In order to more easily assess the relative importance of various covariates on feeling devalued, we fit another logistic regression to model their probability *p* of reporting the topic of devaluation (1 = reported devaluation, 0 = did not report devaluation), using faculty gender (0 = man, 1 = woman), race (0 = white, 1 = person of color), rank (0 = pretenure, 1 = tenured), whether they are in a STEM or non-STEM domain (0 = non-STEM, 1 = STEM), whether they were employed by a high-prestige institution (0 = bottom half of the hierarchy, 1 = top half of the hierarchy), and whether they were current faculty or not (0 = former, 1 = current):


$$\begin{array}{rl} \text{logit}(p) & =\beta_{0}+\beta_{1}[\text{Women}]+\beta_{2}[\text{BIPOC}]\\ & \;+\beta_{3}[\text{Tenured}]+\beta_{4}[\text{STEM}]\\ & \;+\beta_{5}[\text{High prestige}]+\beta_{6}[\text{Current faculty}]. \end{array}$$

Odds for each domain of study (Fig. 4) are in Table S15.

#### Mediation analysis

In order to understand how each topic *T* might independently relate to considering leaving a job, we computed conditional probabilities for all 12 topics among current faculty:


$$\begin{array}{rl} & P(\text{reported topic }T|\text{considers leaving})\\ & \;=\frac{P(\text{reported topic }T\text{ AND considers leaving})}{P(\text{considers leaving})}. \end{array}$$

Current faculty could select the option “I would not consider leaving” in the survey (Section S1B). We computed probabilities separately among men and women current faculty (Fig. 5A).

While these probabilities were calculated independently, a multiple mediation model allows us to examine which topics best explain the relationship between gender and retention while accounting for their potential interrelationships as well as other potentially influential covariates (57). We fit two separate models for current vs. former faculty. In each of the models, the retention outcome variable was defined differently. The model for current faculty estimated the effect of gender on considering leaving a position (Fig. 5B), while the model for former faculty estimated the effect of gender on actually leaving a position (Fig. 5C). We included all 12 topics as well as faculty race, whether they were in a STEM field, institutional prestige, and whether they were tenured in the models.

We used semopy (82) to first define the paths from gender to each of the mediators, then define the paths from the mediators to the retention outcome variable, as well as the direct effect (considering leaving for current faculty, left for all faculty). After defining the model, we initialized and fit it with default parameters from the semopy package (82) to get estimates for a1–a16 and b1–b16, as well as the direct effect c_prime. Additional details and estimates in Section S3C (current faculty: Table S17, former faculty: Table S18).

We emphasize that this mediation framework is used as a descriptive tool to characterize the relative associations between workplace topics, gender, and retention outcomes. Given the cross-sectional and observational nature of the data, these models should not be interpreted as establishing causal pathways. Unobserved confounding, correlated mediators, and measurement limitations all constrain causal interpretation (58, 78).

## Supplementary Material

pgag306_Supplementary_Data

## Data Availability

Open-source code is available on Zenodo at https://zenodo.org/records/22261806. Due to the sensitive nature of the survey data, only binary 0/1 data have been released at https://zenodo.org/records/22262145 indicating whether each person mentioned a topic or not, along with binned identifier variables. Full data including quotations may be available to qualified researchers upon request; interested researchers may contact Katie Spoon at kspoon@stanford.edu or Aaron Clauset at aaron.clauset@colorado.edu.
