# Supplementary material for: Devaluation underlies gendered US faculty departures across disciplines and institutions
Source: PNAS Nexus. 2026 Oct 13;5(10):pgag306. doi: 10.1093/pnasnexus/pgag306 (PMC13659778; doi:10.1093/pnasnexus/pgag306)
Supplement: Supplementary file 1 [file pgag306_supplementary_data.pdf]

Supplemental Materials

Devaluation underlies gendered U.S. faculty departures across disciplines and institutions

Katie Spoon, Joanna Mendy, Maria Martinez, Mirta Galesic, Daniel B. Larremore, Aaron Clauset, Lauren A. Rivera

This PDF contains:

S1. Survey design and analysis

- A. Population
- B. Respondents
- C. Survey questions
- D. Survey data cleaning

S2. Qualitative design and analysis

- A. Codebook
- B. Analysis
- C. Exemplar quotes

S3. Quantitative design and analyses

- A. Regression: Reporting each topic by group
- B. Regression: Reporting devaluation
- C. Conditional probabilities and mediation analysis

S4. Supplementary analyses

- A. Current faculty
- B. Former faculty

S1. Survey Design and Analysis

The following descriptions of methods in sections S1A-S1D largely follow the descriptions of Spoon et al. [1] because both projects are based on the same population, survey and associated data. As a consequence, the descriptions of methods herein follow those of Ref. [1] closely, and in some cases where precision requires it, may be identical.

A. Population

The survey frame originated from a census of U.S. tenure-track and tenured faculty members obtained under a Data Use Agreement with the Academic Analytics Research Center (AARC). The original longitudinal dataset contained annual employment records from 2011-2020 and doctoral degrees for all tenure-track faculty at all 391 doctoral degree-granting institutions in the U.S. We cleaned and annotated the original dataset, resulting in the final population that we sampled from for our survey. For each faculty member in the dataset, we utilized the following information in our analyses:

- **PhD year**: The year in which they received their doctoral degree
- **Discipline** (annotated): STEM/non-STEM, nine domains and 111 fields
- **Prestige** (annotated): The relative prestige of their employing institution

I. Data cleaning

Individual-level cleaning. We filtered the larger census of U.S. faculty, which included temporary faculty. Our frame includes only regular faculty members, i.e., those tenured or on the tenure track with titles of "assistant professor," "associate professor," and "full professor." We filtered out and removed faculty with titles or ranks like "lecturer," "instructor," or teaching professors (all ranks), as well as faculty of unknown rank, and research, clinical, or visiting faculty. In addition, 29,872 faculty members (representing 9.8% of all faculty in our dataset) were missing doctoral degree years and were excluded from our analyses.

We removed outdated employment records. Rarely, some faculty members in the dataset appear as faculty members at multiple universities in a single year. These situations most likely represent a professor who has switched institutions, but whose old employing university (the university that they left) has not yet removed the professor from their public-facing employment records. These are residual records of employment, so we removed them, which removed 0.23% of total employment records. Records of employment preceding events where professors switched institutions were not removed.

Finally, we imputed missing employment records. Rarely, some faculty members disappear from the dataset only to later reappear in the same department they left. While rare, it is important for us to understand and address these events, as our study is interested in gender differences between meaningful attrition events. We consider these situations to be spurious attrition events, and impute employment records for the missing years using the academic rank the faculty member had prior to "leaving". Employment records were not imputed if they were listed at a department with no employment records in the given year. We imputed 1.3% of employment records, affecting 4.7% of people in the dataset.

Department-level cleaning. We removed departments that grew or shrank by more than 50% in a given year. Very rarely, faculty rosters were updated on a delay, with some departments having a relatively large number of faculty added or removed in a given year. Setting a threshold mitigates this artifact of data collection, although changing this arbitrary threshold did not change the qualitative results of the analysis. In addition, we removed departments with less than three unique people across all years, as a department with only one or two people may be a residual record of an old department that has been dissolved.

School-level cleaning. Medical schools were excluded since tenure at those schools often does not guarantee salaries. We de-duplicated the names of departments that were (i) represented in different ways (e.g., "Department of Computer Science" = "Computer Science Department") or (ii) were renamed within the study period (e.g., "USC School of Engineering" = "USC Viterbi School of Engineering").

These steps resulted in a reduced, but more robust dataset. Additional technical details in [2]. We then preprocessed this dataset to add annotations critical to our analysis, described below.

II. Data annotation

Taxonomy annotations. We annotated each department with at least one taxonomic label at two levels: domain and field. This taxonomy allowed us to analyze faculty retention at each level, or to compare patterns between levels. We then annotated each domain of study as a STEM or non-STEM domain.

In cases where a university had multiple departments within the same field, those departments were collapsed into a single unit. For example, Carnegie Mellon's School of Computer Science has seven separate departments. In our taxonomic annotations, all seven departments were annotated as computer science and treated as a single unit in subsequent analyses of computer science.

Some fields are interdisciplinary by nature, with more ambiguous boundaries, and as a result have the potential to conceptually reside in multiple domains. For example, computer engineering could be reasonably included in the domain of mathematics & computing or in engineering. Similarly, educational psychology could be included in the domain of education or in the social sciences. For these ambiguous cases, we grouped each field with the domain that had the largest fraction of faculty whose doctoral university had a department in that domain. In other words, we group fields into domains using the heuristic that fields are best grouped into the domains in which their faculty members are most likely to have been trained.

Prestige annotations. For each employing university, prestige ranks were calculated using the SpringRank algorithm [2,3], which assigns real-valued inferred ranks to institutions (as opposed to ordinal ranks), based on the network of their interactions with each other (specifically, the number of faculty who graduate from and are hired by each institution).

B. Respondents

To construct our survey frame, we selected 29 different fields. We chose this selective strategy because we could not email all faculty in the population described in Section S1A due to financial constraints. Fields were selected across the natural sciences, math and computing, engineering, medicine, social sciences, business, humanities, education, and health (**Table S1**), representing 145,455 current and former tenure-track faculty, comprising 59.3% of the larger dataset (Section S1A). The fields were chosen to cover all of the nine broad domains. Within each domain, a mix of men-dominated fields (e.g., computer science) and women-dominated fields (e.g., nursing) were chosen, to the extent possible. Additionally, we aimed for a mix of large fields (e.g., biology) and medium fields (e.g., information science), and omitted very small fields (e.g., forestry) to avoid small sample sizes. Finally, we selected a mix of both commonly discussed fields in the retention literature (e.g., STEM fields like computer science) and less commonly discussed fields (e.g., education).

Participants fell into four possible target populations: current faculty who did not leave a position anytime after 2009, faculty who left academia, faculty who switched institutions but did not leave academia, and retired faculty (**Fig. S1**).

Participants were invited to participate in the survey through an email invitation, with initial invitations sent from late July 2021 to mid September 2021. Participants were sent two follow-up emails if they had not completed the survey, one week and two weeks after the initial invitation.

Email collection. We attempted to obtain email addresses for all faculty within the selected disciplines in a two step process. First, we utilized a web scraper to make automated web searches. For the faculty whose emails could not be found through this approach, we posted a task on Amazon Mechanical Turk, an online crowdsourcing marketplace. Mechanical Turk workers searched for publicly available email addresses of these remaining faculty members, with the option to indicate that they could not find the email. The authors conducted manual spot-checks to correct errors. Many former academics left forwarding addresses at their old email addresses that we then collected and sent an additional round of emails to. Through this process, email addresses for 50.2% of our full sample were found, resulting in a frame of 73,049 current and

former tenure-track faculty (**Table S1**).

Responses. 73,049 people were contacted, but 1,600 emails failed or bounced, so 71,449 people had the chance to see the email invitation.

Although 11,908 people responded to the survey, 1,470 respondents did not fill out at least 80% of the survey, and 244 respondents were not in our survey frame, either indicating that they were not tenure-track faculty or that they left their positions before 2009, so these responses were removed from the analysis. Out of those who did not fill out at least 80% of the survey and who filled out the demographic information, no significant completion rate differences were found between men and women, or between white respondents and respondents of color.

Additionally, since all of our main analyses include gender, we removed 118 responses from people who did not specify their gender. Since small sample sizes can prohibit reliable conclusions, we also removed responses from 1 person who said they are gender nonconforming, and 4 people who selected that they wanted to self-describe, but did not describe their gender.

After removing these responses, 10,071 people were included in our analysis, so our final response rate was 10,071 / (73,049 - 1600 - 244) = 14.1% (**Table S2**). Overall, our response rate mirrors other online surveys with email invitations conducted in the context of academia [4, 5]. The response rate was higher for former faculty (those who left academia or retired; 34%) than the average (**Table S3**).

The majority of respondents answered the free-text question. **Table S4** shows response rates to this question across groups. In addition, while responses from all faculty who provided a response were coded, a small fraction of faculty (168/6615 = 2.5%) did not mention anything concrete in their response (e.g., "I love everything about my job," coded as "NULL"; see Section S2A), so those respondents were removed from the denominator of the frequency analyses.

Representation. We evaluated the observable differences between the population described in Section S1A, the survey frame, the survey respondents, and the free-text respondents along four key variables: gender, academic rank, STEM vs non-STEM, and prestige (low vs high). The gender variable used was self-reported for survey respondents and algorithmically estimated for the larger comparison population [6].

First, the frame differs in a few key ways from the population, likely due to the non-uniform availability of email addresses online (**Table S5**).

- Men were slightly more likely to be selected into the frame ($\chi^2 = 21$, $p < 0.001$),
- assistant professors were much less likely to be selected into the frame ($\chi^2 = 997$, $p < 0.001$), whereas associate and full professors were more likely to be selected into the frame (assoc: $\chi^2 = 17$, $p < 0.001$; full: $\chi^2 = 494$, $p < 0.001$),
- and faculty employed at higher-prestige institutions (defined in this case as the top 25% of institutions in the hierarchy) were slightly more likely to be selected into the frame ($\chi^2 = 236$, $p < 0.001$).

Second, our respondent group exhibits some differences relative to the survey frame, in that (i) full professors were somewhat overrepresented ($\chi^2 = 10$, $p < 0.01$), (ii) assistant professors were somewhat underrepresented ($\chi^2 = 39$, $p < 0.001$), and (iii) professors from higher-prestige institutions were somewhat underrepresented ($\chi^2 = 91$, $p < 0.001$). In addition, among STEM faculty, men were slightly underrepresented ($\chi^2 = 33$, $p < 0.001$), with no gender differences among non-STEM faculty (**Table S5**).

Finally, those who responded to the free-text question differed in some ways from the survey respondents who did not (**Table S5**). STEM faculty free-text respondents were more likely to be women compared to the frame ($z = 4.8$, $p < 0.001$), but proportions across ranks and prestige did not differ. Non-STEM faculty free-text respondents were also more likely to be women ($z = 7.2$, $p < 0.001$), full professors ($z = -2.3$, $p = 0.02$), and from lower-prestige institutions ($z = 4.4$, $p < 0.001$).

To mitigate these differences in representation, we split our analyses by gender, career age, discipline, and institutional prestige.

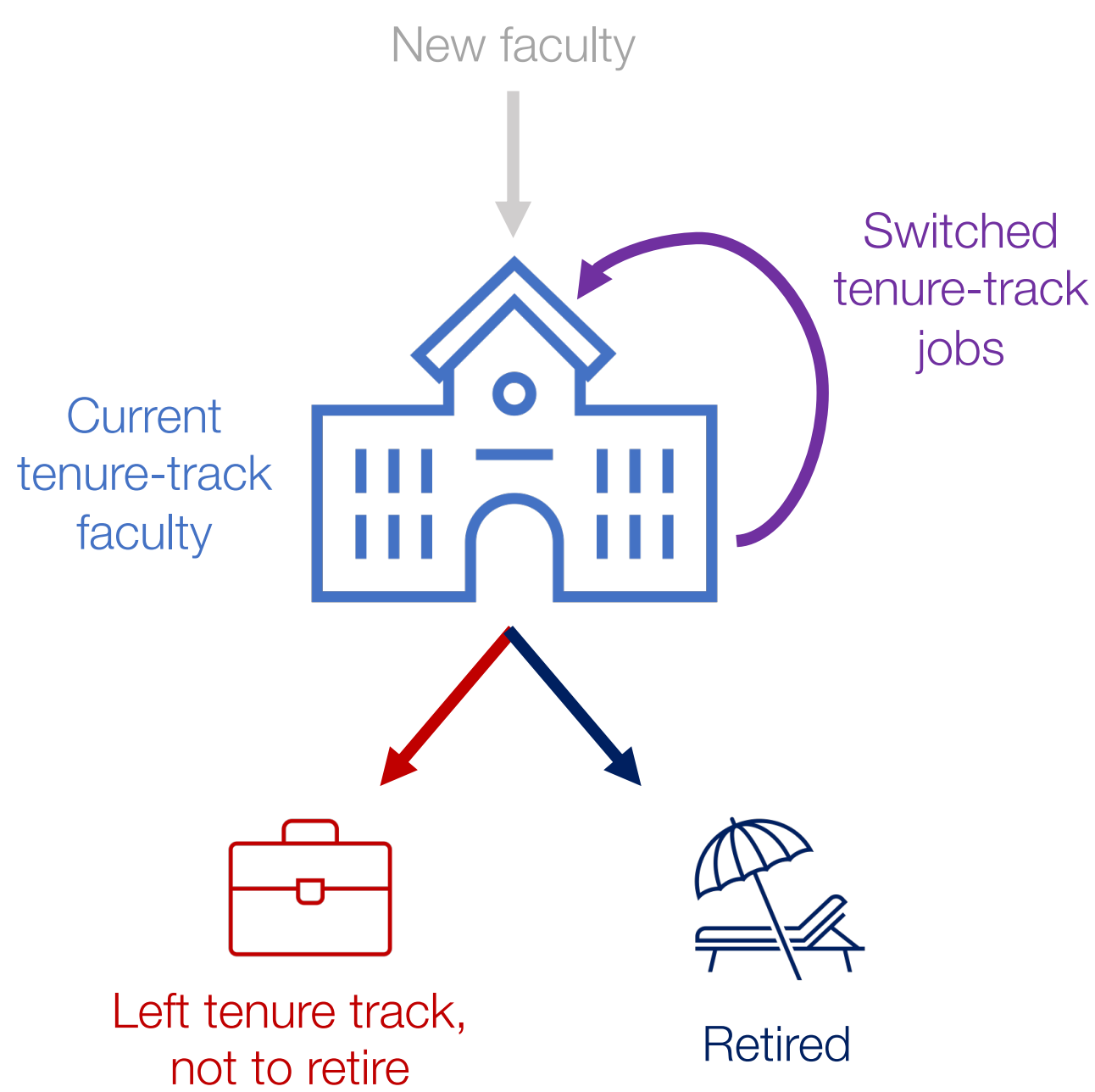

Figure **S1**: **Survey target populations.** We surveyed four target populations: current tenure-track faculty who did not leave a job anytime since 2009, faculty who switched tenure-track jobs, faculty who left the tenure track, not to retire, and faculty who retired. If respondents switched or left multiple times within our time period, we asked them to indicate the first time they switched or left, so for our purposes, these are four mutually exclusive target populations.

Domain	Field	Total Faculty	Emails Found	%
Natural Sciences	Astronomy	2978	1548	52.0
	Biochemistry	6546	2764	42.2
	Biology	9022	4451	49.3
	Biomedical Engineering	3273	1334	40.7
	Biostatistics	2333	955	40.9
	Chemistry	7138	3297	46.2
	Physics	7347	3261	44.3
Health	Nursing	6421	1399	21.8
	Social Work	3393	1347	40.0
Medicine	Epidemiology	2967	1059	35.7
	Pharmacology	3661	984	26.9
	Physiology	3821	1044	27.3
Humanities	History	7458	4305	57.7
	Linguistics	1336	706	52.8
	Philosophy	3668	2081	56.7
Social Sciences	Anthropology	4045	2004	49.5
	Economics	6311	3625	57.4
	Psychology	9119	6533	71.6
	Sociology	4888	2483	50.8
Engineering	Civil Engineering	4519	2530	56.0
	Mechanical Engineering	6677	3774	56.5
Business	Accounting	2978	1683	56.5
	Finance	3413	2007	58.8
	Management	5715	3475	60.8
	Marketing	2728	1464	53.7
Math & Computing	Computer Science	8608	5706	66.3
	Information Science	2173	707	32.5
	Mathematics	8582	5026	58.6
Education	Education, General	3943	1020	25.9
		**145,455**	**73,049**	**50.2**

Table **S1**: Number of faculty members in employment records in each field, and number of faculty whose email addresses could be found. % is the rate email addresses could be found per field.

Domain	Field	Frame	Responses	%
Natural Sciences	Astronomy	1548	275	17.8
	Biochemistry	2764	464	16.8
	Biology	4451	861	19.3
	Biomedical Engineering	1334	205	15.4
	Biostatistics	955	129	13.5
	Chemistry	3297	311	9.4
	Physics	3261	637	19.5
Health	Nursing	1399	183	13.1
	Social Work	1347	169	12.5
Medicine	Epidemiology	1059	175	16.5
	Pharmacology	984	127	12.9
	Physiology	1044	135	12.9
Humanities	History	4305	678	15.7
	Linguistics	706	134	19.0
	Philosophy	2081	314	15.1
Social Sciences	Anthropology	2004	384	19.2
	Economics	3625	353	9.7
	Psychology	6533	1087	16.6
	Sociology	2483	543	21.9
Engineering	Civil Engineering	2530	307	12.1
	Mechanical Engineering	3774	324	8.6
Business	Accounting	1683	215	12.8
	Finance	2007	205	10.2
	Management	3475	355	10.2
	Marketing	1464	181	12.4
Math & Computing	Computer Science	5706	559	9.8
	Information Science	707	119	16.8
	Mathematics	5026	494	9.8
Education	Education, General	1020	148	14.5
	**73,049 - 1600 - 244 = 71,205**		**10,071**	**14.1**

Table **S2**: Number of participants targeted in each field, and number of respondents. % is the response rate per field.

Type of event	N_{total}	$N_{selected-fields}$	$N_{respondents}$	%
Leaving academia or retiring	8,793	4,015	1,387	34%
Switching institutions	22,064	12,564	1,489	12%
Current faculty	203,586	54,626	7,195	13%

Table **S3**: The number of unique faculty who experience each type of event (leaving, switching, or staying) in the population, the number of unique faculty who experience each type of event in our survey frame, and the number of survey respondents. % is the response rate per type of move.

Type of event	N_{total}	$N_{selected-fields}$	$N_{free-text}$	% frame	% respondents
Leaving academia or retiring	8,793	4,015	1,102	27%	79%
Switching institutions	22,064	12,564	1,095	9%	74%
Current faculty	203,586	54,626	4,340	8%	60%

Table **S4**: The number of unique faculty who experience each type of event (leaving, switching, or staying) in the population, the number of unique faculty who experience each type of event in our survey frame, and the number of survey respondents **who answered the free-text question**. % frame is the free-text response rate per type of move compared to the survey frame, and % respondents is the free-text response rate per type of move among survey respondents.

		M	W	NB	Asst	Assoc	Full	High-prestige	N
**STEM**	Frame	78.7	21.3	–	14.2	25.1	60.7	56.6	35,201
	Population	77.3	22.7	–	23.5	23.8	52.6	51.0	63,156
		***	***		***	***	***	***	
	Respondents	74.9	24.7	0.4	10.8	26.0	63.2	49.1	5,029
	Frame	78.7	21.3	–	14.2	25.1	60.7	56.6	35,201
		***	***		***	ns	**	***	
	Free-text	72.5	27.0	0.5	11.0	25.3	63.7	48.4	3,242
	No free-text	79.2	20.6	0.2	10.5	27.4	62.2	50.3	1,787
		***	***	ns	ns	ns	ns	ns	
**Non-STEM**	Frame	56.6	43.4	–	17.3	30.6	52.2	57.5	36,004
	Population	54.9	45.1	–	27.4	29.1	43.5	45.1	58,510
		***	***		***	***	***	***	
	Respondents	56.0	43.7	0.3	14.8	29.8	55.3	52.5	5,042
	Frame	56.6	43.4	–	17.3	30.6	52.2	57.5	36,004
		ns	ns		***	ns	***	***	
	Free-text	51.9	47.6	0.5	15.4	30.5	54.1	50.0	3,295
	No free-text	63.3	36.5	0.2	13.7	28.7	57.7	56.9	1,747
		***	***	ns	ns	ns	*	***	

* $p < 0.05$; ** $p < 0.01$; *** $p < 0.001$

Table **S5**: Demographic attributes of faculty in STEM vs non-STEM fields. W = women, M = men, NB = non-binary, Asst = assistant professor, Assoc = associate professor, Full = full professor. The proportion in high-prestige institutions is calculated by selecting faculty who were in the top quarter of the prestige hierarchy. Proportions are reported for the survey frame versus population and the survey frame versus respondents, with statistical significance calculated using χ^2 two-tailed tests. Proportions are also reported for survey respondents who answered the free-text question compared to those who did not, with statistical significance calculated using two-proportion z tests.

C. Survey questions

This survey was approved by the University of Colorado Boulder Institutional Review Board (protocol 21-0293) and conducted online through the Qualtrics survey platform. Participants were asked the following questions, in the following order:

1. Select one:
   - I am currently tenure-track/tenured faculty at a PhD-granting university in the U.S.
   - I am a former tenure-track/tenured faculty at a PhD-granting university in the U.S.
   - I have never had a tenure-track/tenured faculty job at a PhD-granting university in the U.S. (excluded from survey)
2. For those who said they were former faculty: Did you LEAVE a tenure-track/tenured job at any point between 2009-2021?

   NOTE: "Leaving" could be voluntary or involuntary (let go), and includes leaving for another tenure-track/tenured job, another academic non-tenure-track/tenured job, a non-academic job (for example: industry, government, self-employment), retirement, or leaving the workforce.

   I left my most recent tenure-track/tenured faculty position:
   - Before 2009
   - In or after 2009

   This survey is intended only for former tenured or tenure-track faculty in the U.S. who left their job in 2009 or later. If you accidentally selected the wrong answer, you can correct it above and continue. Otherwise, you may exit the survey now.
3. What is your gender? [required]
   - Man
   - Non-binary
   - Woman
   - Prefer not to say
   - Prefer to self describe: [–free text box–]
4. What is your race? Select one or more responses. You are welcome to add your origin in the text box. [required]
   Note: We are using the U.S. Census racial categories for comparison to the general population. We are aware these categories are reductive and may not describe you well. Please use the text boxes as needed.
   - ☐ American Indian or Alaska Native: [–free text box–]
   - ☐ Asian: [–free text box–]
   - ☐ Black or African American: [–free text box–]
   - ☐ Hispanic, Latino, or Spanish origin: [–free text box–]
   - ☐ Native Hawaiian or other Pacific Islander: [–free text box–]
   - ☐ White: [–free text box–]
   - ☐ Some other race or origin: [–free text box–]
   - ☐ Prefer not to say
5. Current faculty: Do you have children?
   Former faculty: At the time you first left a tenure-track/tenured position, did you have children?

- Yes
- No
- Prefer not to say

6. How many, and when were they born?
   - How many? Enter the number of children you have: [–free text box–]
   - When were they born? List the years they were born separated by commas: [–free text box–]
7. Please answer the following questions about your terminal degree (your PhD or other degree that qualifies you to be tenure-track faculty).
   - Graduation year [dropdown]
   - Terminal degree (e.g., PhD) institution: [dropdown] If your institution is not listed, select "Other" (Other: [–free text box–])
8. Current faculty: What is your current position in academia? (Other: [–free text box–])
   - Assistant Professor
   - Associate Professor
   - Full Professor
   - Emerita/us
   - Unranked
9. Current faculty: Current Institution [dropdown] (if not listed, select "Other")
10. Current faculty: My primary academic unit (department, or similar) is: [required]
    If not listed, select "Other" (Other: [–free text box–])
11. Former faculty: If you have left multiple tenure-track/tenured faculty positions over your career, please answer the following questions only about the first position you left, within the years 2009-2021.
    - I left in year: [dropdown 2009-2021]
    - (Select one) My rank at the time was:
      - Assistant Professor
      - Associate Professor
      - Full Professor
      - Emerita/us
      - Unranked
    - I left institution (if not listed, select "Other"): [dropdown] (Other: [–free text box–])
    - My primary academic unit (department, or similar) was: [required]
      If not listed, select "Other" (Other: [–free text box–])
    - (Select one) Reason for leaving: [required]
      - I left voluntarily (for example: to leave academia, to switch institutions, to retire, to leave the workforce)
      - I was denied reappointment prior to the tenure evaluation
      - I was denied tenure
      - Other [–free text box–]
12. Current faculty: Think about the reasons that might lead you to consider leaving your position (check all that apply):

    ☐ I am unhappy, stressed, or otherwise less than satisfied with my current position.

☐ I am drawn to, excited by, or otherwise attracted to a different position.

☐ I would not consider leaving.

13. Current faculty: If I were to leave my current position, it would probably be to:

    ☐ Switch academic jobs (e.g., to a new institution)

    ☐ Leave academia for another job (e.g., for industry, government, start-up, etc.)

    ☐ Leave the workforce (e.g., retire or take a break from working)

14. Current faculty: Tell us your story: what needs to be different in order to reduce the stress you feel in your position?: [–free text box–]
    Former faculty: Tell us your story: what needed to be different in order for you to stay in your position?: [–free text box–]
    Note: We hope to learn from personal narratives and qualitatively analyze responses into categories of field-specific solutions for faculty retention, but will not publish direct quotations without asking you for explicit followup permission.

D. Survey data cleaning

During data analysis, there were several data cleaning choices we made that re-classified several groups of responses in order to be as inclusive as possible and as clear as possible. However, no responses were removed during data cleaning.

Gender. 3 out of the 6570 men in our survey indicated they are transgender men, 1 out of the 3480 women in our survey indicated they are a transgender woman, and 1 out of the 3480 women in our survey indicated they are a woman and non-binary. While these 5 individuals used the "prefer to self-describe" box in order to describe their gender, we also included them in the binary gender categories that they mentioned in their descriptions. Including participants who self-reported these additional aspects of their gender identities into "men" and "women" categories allows us to respect their status as men and women [7].

Race. Respondents were given checkboxes corresponding to the U.S. Census race categories and asked to select all the categories they identified with. The options were: "American Indian or Alaskan Native", "Asian", "Black or African American", "Hispanic, Latino, or Spanish origin", "Native Hawaiian or other Pacific Islander", "White", "Other". These categories alone cannot encompass someone's entire racial identity, and we look forward to future survey methods that allow respondents to better represent their identity.

For each racial category a respondent identified with, they were included in that group. Most people only selected one group, and 525 respondents (5.2% of respondents) selected more than one. People who indicated that at least one of their racial identities was non-white were considered people of color, representing 1,747 respondents (17.4% of respondents).

Emerita/us faculty. Respondents were asked to indicate whether they were current or former faculty, along with their academic rank. Respondents who indicated that they are Emerita/us faculty were classified as retired, even if they said they were current faculty members, accounting for 71 respondents (0.7% of respondents).

Considering leaving. We marked current faculty as *considering leaving* if they selected one or both of these options. They were only marked as *not considering leaving* if they explicitly selected "I would not consider leaving."

2. Qualitative Design and Analysis

A. Codebook

Qualitative analyses of free-text data are typically conducted either (i) deductively using a top-down approach starting with a set of predetermined, theoretically grounded themes into which the responses are categorized [8], (ii) inductively sorted into themes that are identified from the responses using a bottom-up approach [9], or (iii) through a combination of both approaches, known as an abductive approach [10].

We analyzed responses to these questions separately for current vs. former faculty through three rounds of inductive qualitative coding [9]. In the first round of coding, we began by developing codebooks of primary (i.e., top-level) and secondary codes for current and former faculty separately; each codebook was based on analysis of a test set of 100 responses and was refined through several rounds of testing and inter-rater reliability checks (**Table S6**). Three of the authors then used these codebooks to hand code all responses ($N = 6,615$), with two authors coding each response. The team met regularly to discuss code disagreements, and a mediator would decide when both raters did not agree [11]. After completing this first coding round (the first full pass through all responses), we ran frequency analyses to determine which categories displayed the largest gender differences in reports. The secondary codes of service, informal climate, mistreatment, and formal evaluation practices were most gendered.

We then conducted a second round coding to capture themes in faculty experiences within these four secondary codes. In this round, we followed the same procedure as in our first round of coding all responses: we developed a codebook of tertiary codes, also based on several rounds of testing on small test sets and inter-rater reliability checks (**Table S7**). This process resulted in several conceptual and structural changes to our initial codebook.

- **Work-related**: The service category split into professional service and excerpts about busywork
- **Organizational**: Formal evaluation split conceptually into evaluation metrics (organizational) and unfair treatment in evaluation, or formal devaluation (relational). Evaluation metrics includes four tertiary codes, and is grouped under administrative policies.
- **Work-life balance**: Codes for location and personal matters (e.g., divorce, illness) were added
- **Relational**: Informal climate was split into (i) devaluation, with tertiary codes of formal and informal (including mistreatment), and (ii) the department environment, with tertiary codes of cohesion, morale, and demographic representation. Ineq. workload was split three tertiary codes.

We then hand-coded all text passages mentioning these secondary codes with their new applicable secondary and tertiary codes. After completing this second coding round, we ran frequency analyses of the codes, which showed devaluation was the secondary code with the largest gender difference.

Finally, to capture themes and sources of variation within the secondary code of devaluation, we conducted a third and final coding round of quandary codes, following the same method, to capture the various ways that women, men and non-binary faculty discussed devaluation at work (**Tables S8-S12**).

In order to build trust with participants and to disclose our intended use of potentially sensitive information, when respondents were taking the survey the research team indicated that we would not share their quotations without seeking their explicit followup permission. Once we had selected an initial set of exemplar quotations, we emailed participants their quotations and asked for their permission. Most respondents gave us permission to use the entire quotation (82.5%), a few opted to edit their quotations to remove identifiable information (7.5%), and one respondent did not give us permission to use their quotation due to safety concerns (2.5%). The rest did not respond to our requests or emails bounced (7.5%).

We then identified additional quotations and sought permission to present those in place of the initial quotations we did not receive permission for. When reporting quotations in our results, we default to including their gender, race (Asian, Black, Hispanic, or white), academic rank (pre-tenure or tenured), and field of study, including other identifiers such as parenthood status where relevant. However, during the quotation permission process they had the option to ask us to use a more coarse description of their identity when presenting their quotation (e.g., “woman of color” instead of their race, or “STEM”/“non-STEM” instead of their field). If a respondentâĂŹs quotation was particularly sensitive, we would use a more coarse description of their identity even if they did not request it.

Themes		Description
Primary	Secondary	
**NULL**		Do not code if they do not mention any stressors
**Work-related**		
Research	Funding	Difficulties obtaining funding, issues with the funding system (e.g., the types of research or expenses that are funded)
Research	Peer Review	Relates to the peer review process of publishing papers or getting grants
Research	Research Group	Relates to the hiring, managing, training, supervision and quality of research assistants (students and postdocs) working in their research group or lab
Teaching		Teaching load, support/resources for teaching, students
Service		Administrative work (i) to the institution (e.g., serving on committees) or (ii) to the broader community (e.g., reviewing, conference organizing)
**Organizational**		
Leadership	Departmental	Specifically mentions their department chair or department head
Leadership	Institutional	Mentions administrators, institutional practices, communication, choices and decisions made by the university
Resources		Administrative support, staff, and programs or services (which typically require staff to run)
Salary		Issues with salary or benefits
Administrative policies		Mentions a specific, existing policy that they have a problem with (e.g., tenure clock stops, parental leave, sabbaticals, 2-body problem)
Formal evaluation		How people evaluate or view their work, including metrics, pressures, expectations for productivity, and ambiguous tenure processes
**Work-life balance**		
Caretaking		Caretaking responsibilities for children, partner, parents, etc.
Workload		Relates to the time constraints someone has due to the job. This also includes mentions of a poor work-life balance climate in their departments, where it is not acceptable to take time off.
**Relational**		
Inequitable work		The inequitable distribution of administrative service, informal duties or responsibilities among colleagues or between faculty and administrators
Informal climate		How people view them as a person (not their work), and how their colleagues interact with them
Mistreatment		Includes bias, harassment, prejudice, discrimination, and other kinds of mistreatment, either localized or systemic

Table **S6**: Initial codebook used to categorize free-text responses.

Themes			Description
Primary	Secondary	Tertiary	
**NULL**			Do not code if they do not mention any stressors
**Work-related**			
Research	Funding		Difficulties obtaining funding, issues with the funding system (e.g., the types of research or expenses that are funded)
Research	Peer Review		Relates to the peer review process of publishing papers or getting grants
Research	Research Group		Relates to the hiring, managing, training, supervision and quality of research assistants (students and postdocs) working in their research group or lab
Teaching			Teaching load, support/resources for teaching, students
Service			Administrative work (i) to the institution (e.g., serving on committees) or (ii) to the broader community (e.g., reviewing, conference organizing)
Busywork			Paperwork, pointless meetings, trainings, or other "busywork"
**Organizational**			
Leadership	Departmental		Specifically mentions their department chair or department head
Leadership	Institutional		Mentions administrators, institutional practices, communication, choices and decisions made by the university
Resources	Services		Administrative support, staff, and programs or services (which typically require staff to run)
Resources	Salary		Issues with salary or benefits
Admin. policies	Evaluation metrics	Unclear	Confusion about how performance is evaluated in their organization
Admin. policies	Evaluation metrics	Unbalanced	Feeling that certain types of work are undervalued (e.g., service) or overemphasized (e.g., research) in departmental evaluations; feeling that their research topic is not valued in their department or institution
Admin. policies	Evaluation metrics	Unreasonable	Feeling that expectations in their department or institution are excessive or misaligned with rewards
Admin. policies	Evaluation metrics	Other	Desire for more recognition or appreciation from their department or institution without specific explanation
Admin. policies	Specific policies		Mentions a specific, existing policy in their organization that they have a problem with (e.g., tenure clock stops, parental leave, sabbaticals, 2-body problem)
**Work-life balance**			
Caretaking			Caretaking responsibilities for children, partner, parents, etc.

Workload			Relates to the time constraints someone has due to the job. This also includes mentions of a poor work-life balance climate in their departments, where it is not acceptable to take time off.
Location			Relates to the physical location of their position, including being far from family, living in a state with different political values, cost of living, etc.
Personal matters			Divorce, illness, etc.
**Relational**			
Devaluation	Formal	Procedural unfairness	Perceived unfair treatment in formal evaluations due to identity or favoritism
Devaluation	Informal	Exclusion	Feeling excluded, isolated and lacking supportive professional relationships
Devaluation	Informal	Mistreatment	Experiencing harassment or microaggressions
Devaluation	Salience		Mentions that an aspect of identity is treated differently or misunderstood by their colleagues. Gender, race, parental status, and other identity (e.g., social class, sexual orientation, disability, etc.) are coded separately.
Department Environment	Cohesion		Feelings of togetherness/connection; that they are all working towards the same goal/mission. A lack of cohesion can be marked by some departmental conflict (in-fighting)
Department Environment	Morale		An observation about how the individual and others feel in their environment (implying everyone feels this way), including energy, job satisfaction, attitude, and outlook, including general group burnout.
Department Environment	Demographic underrepresentation		Mention a lack of demographic diversity in their departments.
Department Environment	Other		Mention a toxic/bad environment without further elaboration
Inequitable workload	Workload		The inequitable distribution of administrative service, informal duties or responsibilities among colleagues or between faculty and administrators.
Inequitable workload	Identity		The inequitable distribution of administrative service, informal duties or responsibilities, where individuals are pressured take on more role due to gender, race, sexual orientation, etc.
Inequitable workload	Junior/senior		The inequitable distribution of administrative service, informal duties or responsibilities, where either junior faculty take on more work than senior faculty or senior faculty take on more work than junior faculty.

Table **S7**: Final codebook used to categorize free-text responses.

B. Analysis

We made several careful design choices during coding and in reporting our qualitative results in an effort to maintain replicability and transparency.

Inclusion and exclusion criteria for devaluation. *Formal devaluation* was defined as perceptions of a lack of fairness in local decisions (departmental or institutional) related to evaluation (tenure, promotion, salary, retention offers), the distribution of resources, internal funding decisions, etc., on the basis of X, where X is one of 5 categories, coded separately; gender, race, parental status, other identity (e.g., social class, sexual orientation, disability, etc.), other general (not based on identity). To be coded as forma devaluation, they must have included **a comparison between two groups**, where the respondent reports receiving unfair treatment compared to the other group. There were two main ways this was reported: (1) if evaluation was not based on merit, but identity (e.g., younger male colleagues who were less successful got promoted but not me) or politics (e.g., someone on the committee didn't like me so I wasn't promoted), or (2) if evaluation was not equitable (e.g., did not include the appropriate accommodations for a single parent), including based on type of work (e.g., the under-emphasis of service performed by women). This code includes both individual instances and systemic (e.g., noticing gender pay gaps in their department).

Informal devaluation consisted of two separate tertiary codes: (1) exclusion - feeling excluded, isolated and lacking supportive professional relationships, and (2) mistreatment - experiencing harassment or microaggressions. People who felt excluded mentioned a lack of positive discrete social relationships such as collaboration, mentoring, and supportive interactions. They reported not having people who provide emotional support (empathy, trust, caring), informational support (advice, suggestions, information), or people to share in the process of generating ideas. They usually reported lacking these relationships from specific people (colleagues, chairs, etc). In contrast, mistreatment included explicit perceptions of bias, harassment, prejudice, discrimination, and microaggressions (localized or systemic) on the basis of X, where X is one of 5 categories, coded separately; gender, race, parental status, other identity (e.g., social class, sexual orientation, disability, etc.), other general (not based on identity). This category could include informal evaluation from colleagues, but not formal, discrete evaluative moments (i.e tenure, promotion).

Gender gaps as differences rather than ratios. The focus of our analysis is not based on the main stressors women faculty experience and the main reasons they leave, but rather, the most *gendered* experiences and reasons. The topics with the largest gender gaps represent the aspects of academia that need to be improved most to ameliorate any gendered stressors that women face on top of universally stressful, non-gendered topics. A "gender gap" can be calculated in multiple ways. The two simplest ways are (i) the difference between the frequency at which women reported a topic and the frequency at which men reported a topic (subtraction) and (ii) the ratio between the frequency at which women reported a topic and the frequency at which men reported a topic (division).

Ratios are perhaps the most appropriate way to show the most gendered difference, because they do not take into account the overall frequency at which a topic appears. We found that the most gendered ratio was for the inequitable distribution of work (reported by 12.3% of women and 2.7% of men, among current faculty; $z = 11.9, N = 4,274, p < 0.001$, a difference of 9.3% and a ratio of 4.1), the second was caretaking responsibilities (reported by 11.9% of women and 3.0% of men, among current faculty; $z = 11.5, N = 4,274, p < 0.001$, a difference of 8.9% and a ratio of 3.9), and the third was devaluation (reported by 38.4% of women and 15.6% of men, among current faculty; $z = 16.9, N = 4,274, p < 0.001$, a difference of 22.8% and a ratio of 2.5).

Although the inequitable distribution of work and caretaking responsibilities displayed higher gendered ratios than devaluation, they were much less prevalent among faculty than devaluation was. Since devaluation still exhibited one of the largest gendered ratios, but also the largest gendered difference by far, we chose to further investigate devaluation as the basis of our analysis. In addition, the inequitable distribution of work and caretaking responsibilities among women faculty have been well-documented in the literature, while devaluation among women faculty has generally not been investigated as much.

General vs gendered devaluation. We coded data separately as general devaluation (not based on identity) or identity-based devaluation, separately for informal devaluation, formal devaluation, and salience.

In our final results, we group all devaluation together, because it is possible that a response we coded as general devaluation was actually identity-based, but the respondent excluded their identity in their response. This represents a limitation of a text analysis, compared to an interview study where we could ask them to clarify if the devaluation was identity-based or not.

We also did not view the respondents' self-reported demographic information while coding in order to remain neutral and not make inferences about the devaluation they experienced. Hence, faculty of all genders, including men, reported gender-based devaluation, and faculty of all genders, reported general devaluation that was not identity-based. In order to demonstrate the difference between general and gendered devaluation, we have included several examples below.

Gendered formal devaluation: "No matter how hard you worked and how great your proposal is, you received the CAREER award because you were a woman or minority." - white tenured woman in engineering

General formal devaluation: "The tenure system needs to be abolished. Promotion needs to become based on merit, accomplishment and productivity, not popularity and politics. Faculty shouldn't have to apply for promotion. Rather, promotion should be automatically granted when faculty have achieved predetermined levels of success and accrued experience." - white tenured man in physics

Gendered informal devaluation: "Some of my male colleagues harassed me, ignored me, derided my work or made the department a hellish place to work and teach, as I knew they were also harassing or even sexually abusing students, but I could never get any of them convicted. I survived, but not without rage and scars." - white tenured woman in english literature

General informal devaluation: "We need administrators who actually care about education rather than their own next position, and who listen to faculty and treat us as experts rather than expendable employees. Meanwhile, we also need faculty who treat each other with respect and are willing to act as team members. Unfortunately the current state of academia means that no one has any incentive to do these things, and there is no accountability for either admins or tenured faculty." - Asian tenured man in mathematics

C. Exemplar quotes

A. Higher expectations

The expectations for women lucky enough to be full professors are extraordinarily high. - white tenured woman, anthropology

I have to do twice as much work for it to get any recognition at my university. I've submitted two [book] manuscripts and am about to publish one of them with top-notch press, while my male colleagues get rewarded for being mediocre. - tenured woman (race unspecified), history

B. Fewer resources

The male assistant professor I was hired to replace had funding for graduate research assistants...written into his contract, but when I asked for that I was told that [my university] didn't do that anymore. Then a male colleague who came in [after me] - yep, you guessed it! He got that same funding. I succeeded without this - I got tenure. But the amount of added stress, and just the general feeling that the school does not value me in the way it values those male colleagues, is extremely disheartening. - white tenured woman, linguistics

I am sick of publishing more and better than my male colleagues and still earning only 75% of what they earn. It's not just publishing...I have the best track record of graduate student placement in my department. I have earned more professional awards and formal recognition than all of my peers in my department. I constantly feel like it doesn't matter what I do, I will never be good enough. It is emotionally exhausting and affects my productivity. - Asian tenured woman, economics

C. Achievements are unnoticed or devalued

*Academia prides itself on being an incubator for creativity and innovation, but then values people byâĂęproblematic intangible factors (visibility, "fit", prestige, connections, "golden" status). I [had] a lot of work-related stress from realizing how little my colleagues thought of me...People in academia treat each other, and critically, *evaluate* each other, differently depending on how much they think they are worth.* - Hispanic tenured woman, psychology

I was the first female faculty member in my department and received several comments suggesting that I would benefit from being a woman - like it was easier to receive funding. There was a lot of doubt about my grant proposals being funded for their own merit. - white tenured woman, chemistry

Our dean recognizes [our new male colleague] on sight and knows about his research, but not the female faculty, despite being hired before [him]. Additionally [he] has been publicly recognized for his work (publications, presentations), while my female colleague received a prestigious grant that was not mentioned at all. - Hispanic pre-tenure woman, social work

D. Unbalanced service loads

There is a lot of stress on us to "represent the Black faculty" and support Black students - while still trying to do all the other things every faculty member does...So we again and still have to do everything twice as well and twice as much, especially if we care about the students...There are very few faculty who work with Black students...And the administration is friendly and seemingly sympathetic but doesn't give or put the necessary resources into reducing these stressors or supporting those of us who take this onâĂęWe faculty of color and women, need to be recognized and rewarded for all the extra time and energy we put in to advising students, keeping the college honest, [and] addressing institutional racism in all its manifestations. - Black tenured woman, psychology

While technically I could say "no" to those events and committees (and many non-minority mentors had told me to say "no" as well), the implications of saying "no" too often remains unclear at this point, and this is especially stressful for a tenure-track (not yet tenured) faculty. - Asian pre-tenure woman, engineering

E. The bar is higher outside of work

I have two kids under four and have been home with them with no childcare since March 2020. This isn't a sustainable work environment, and my productivity has suffered greatly. Yet the standards for productivity in our field and for promotion (e.g., grants, publications) don't seem to take this context into account. I will have the same expectations for promotion as my childless male colleagues—many of whom are thriving in this work-from-home environment—even though our life circumstances are very different. - white pre-tenure mother, psychology

Table **S8**: **Gender-based formal devaluation.** Exemplar quotations from women faculty describing formal devaluation in their jobs, reporting feeling like (A) they are expected to meet higher expectations (B) with fewer resources, (C) fewer rewards, while balancing (D) higher service loads and (E) greater caretaking responsibilities outside of work.

**A. Subtle exclusionary behaviors**
It's 1000 cuts. From colleagues not saying hi to you when you say hi to them. Or knowing that a group of guys regularly goes to lunch and being brave enough to ask them to come get you but then have them not follow through…To have male department chairs excited that the university policy makes it so that I could take my parental leave at any point during the academic year so I could teach my regular class, even though…physically I cannot even exercise at that point but standing for multiple hours to lecture is normal. To be given new preps as punishment for fighting back against the original teaching assignment which was clearly a sexist application of the university parental leave policy. To have men interrupt and aggressively question my knowledge base. To never be given the benefit of the doubt…To get no mentoring despite a formal mentor. To do 90% of the work on research papers but share credit as white males just free ride. - white pre-tenure woman, business
As a woman, I often feel that people in my institution are trying to impose boundaries on my life choices…I've gotten endless amounts of unsolicited advice on when I should start my family, including being told that women "aren't encouraged to have children before tenure", that maternity leave cannot be more desirable or else "women would never go up for tenure" or would "keep having kids to avoid going up for tenure." No one has ever mentioned to my husband when they feel he should consider having kids relative to his tenure clock. Then there are the micro-aggressions, like being told to smile more. Again, no one has ever walked up to my husband during a boring faculty meeting and told him to smile. - Black pre-tenure woman, business
**B. Subtle exclusionary behaviors are particularly salient for marginalized women**
I was advised to hide the fact that I ever spent time on childcare and enjoying being with my kids. I was told to hide all this in order to get tenure, so I would mark "research" or "fieldwork" on my calendar rather than anything to do with children. I resented this greatly and it added to huge pre-tenure stress. I had a male colleague, however, who would sometimes bring his baby to campusâĂŤpeople applauded him for his gender sensitivity, but I think women who bring young children to the university context still risk being denigrated. - white tenured mother, anthropology
The environment is toxic and extremely racist. Constant discrimination that is subtle and awful. Example - [I was] promoted [3 years ago] and [my] nameplate was not changed. A man was promoted last month and has a new name plate. In fact, all people promoted since me have a new title on their door. Mine does not. This is intentional and awful. - Black tenured woman, social work
Throughout my career, issues of sexism have been pervasive in academia. Academia, despite the rhetoric of interest in addressing these issues, needs to work harder…One issue that pervades my interactions in academia is not being valued as an academic. My husband, who is also an academic, receives far greater respect than I do from my colleagues in the department, in the university, and in professional organizations. Being a woman of small stature with nearly white hair, the problem has become more prominent as I age. - white tenured woman, psychology
**C. Overt mistreatment**
I came to my university as 1/2 of a romantic partnershipâĂęWhen I ended the relationship…multiple high-level members of my department shunned me…Almost all of these senior faculty are close friends with my ex-boyfriend. One of [them] offered to be my professional mentor…and then began acting inappropriately. When I rejected his advances, he sexually assaulted me and then withdrew all professional networking opportunities he had previously offered me…When I came up for tenure, despite holding [a very prestigious and large national research grant], the tenure committee heavily stacked with my ex-boyfriend's buddies voted unanimously against my tenure and unanimously for his. He had no grants…and a comparable publication record to mine. Thankfully, all other levels of university review…supported my tenure whole-heartedly. - woman of color
The university system is toxic and abusive. I was sexually harassed while on maternity leave and my department chair still believes my harasser over me. Toxic white males are always protected. - white tenured woman, anthropology

Table **S9**: **Gender-based informal devaluation.** Exemplar quotations from women faculty describing informal devaluation in their jobs, reporting (A) a range of subtle exclusionary behaviors (B) that are particularly salient for marginalized women, such as mothers, women of color, and older women, as well as (C) overt mistreatment.

A. Indifferent institutional response

While few people actively worked to derail my career, the indifference of my so-called colleagues and the administration to the toxic work environment was thoroughly disheartening. ["Cover your ass"] was the name of the game, rather than what we can do to improve things. While there has been more talkâĂęabout DEI at my institution, progress is negligible. Given the glacial speed at which universities change, I don't expect much. - Hispanic tenured woman, chemistry

In nearly twenty years of being a faculty member, it is disheartening to see the needle barely budge on [the unequal and gendered service burden]. It's demoralizing. Universities need to value all this time and labor and recognize its central importance with more than the occasional pat on the back. - white tenured woman, history

The tenure track is extremely stressful by itself…However, this stress is compounded by a hierarchy that disadvantages women and people of color, who have to put up with misogyny and racismâĂęwhile on the tenure track, something our white, male colleagues do not. This is true of any job…but it is worse in academia because the hierarchy protects people from criticismâĂęI have seen little evidence that recent pushes for racial and gender justice have had an effect on the academy because its structure protects against making wholesale changes. - white tenured woman, history

B. Performative institutional response

I am the only woman in my department and while I have prominence in my profession, I am paid less than my male peers, even some junior to me. I am overworked in service…Most importantly, I have asked for all of these matters to be addressed and have been declined, the dean not even [pretending to be] disturbed by my inequitable conditions and pay. I am stressed by the hypocrisy of the institution, which regularly touts its commitment to equity so I would (minimally) like them to shut up about that. - white tenured woman, philosophy

As I've begun to speak up for myself and question what I see as unfairness, I sense I am seen as difficult…But I'm repeatedly told how "valued" I am, that people "want me to feel supported," phrases which I now interpret as a sort of getting the bureaucratic finger: "Too bad! Not my problem!" I struggle in an expletive-free way to appropriately describe the situation…What I cannot make peace with is that SO many hours, so much of my timeâĂŞin meetings, discussions, required forms to fill out, documents to submit, boxes to check, trainings to takeâĂŞall dedicated to this performance of fairness and equity, have been stolen from me, while these basic facts of my existence at the institution belie all the sanctimony. If it is going to be unfair, if merit isn't what determines raises…if the only "path forward" for a woman like me is to just be fine with getting less, then stop wasting my time with these performances about fairness and spare me all the patronizing, condescending sanctimony about commitments to justice and equity. - white tenured woman, history

After [I had spent nearly 2 decades] building…our teacher education program, hand picked women of color or white women [were] placed in positions of influence to perpetuate a more compliant environment. Where there would be a robust social justice focus there is now a sanitized "inclusive excellence" focus, where white women are the face of equity…and there is no real positive social change in our program. - Black tenured woman, education

In a word: hypocrisy. At my university and in my college, progressive values and tolerance of diversity are performative. As a lesbian, I have dealt withâĂęmicroaggressions for [decades]. [Recently]…I learned that I was earning [tens of thousands of dollars] less than my peers. When I spoke with my dean about a raise, he told me that "the university would go broke if it tried to pay you what you should be getting"…working among people who profess yet don't act on progressive values is demoralizing. - white tenured woman, social sciences

C. No institutional response to mistreatment

The infrastructure openly attacks women and minorities. Yet they claim to be [a highly] diverse school…A senior prof (male) was openly caught trying to steal one of my awards and then sabotaged my lab when he was caught and no one did a thing. - tenured woman of color, social sciences

[I told] a dean about the unwelcoming climate and was told to go through a bunch of hoops, which I did and took lots of time. Then, [I was] told that the best they could do about sexist tenured faculty members is to "nudge" them to be nicer. - white pre-tenure woman, business

Bullying and intimidation are rarely recognized but exist within departments. I was harrassed (and will be at the start of the semester) by a male faculty member in my department. The department and dean refuse to act because I have not been directly threatened. - white tenured woman, sociology

Table **S10**: **Lack of institutional response.** Exemplar quotations from women faculty who reported gender-based devaluation to their institutions, who often (A) respond with indifference or (B) performative rather than concrete action, (C) often donâĂŹt respond to reports of mistreatment.

A. Leaving because of devaluation

My department and my field both systematically discriminate against women. I found out recently IâĂŹm the lowest paid in my department despite out-pacing many of my male peers in productivity and being here for...years. IâĂŹve had grants rejected due to "lack of leader potential" despite being consistently asked to lead community efforts in the field. And when I bring up evidence of issues like these, more times than not IâĂŹm told my problems arenâĂŹt real problems. No matter what I do, the pervasive lack of support for women at my institution and in my field continuously sets me up for failure and because of that IâĂŹm looking for other jobs. - white pre-tenure woman, engineering

An open call for a new director was held [for our center]. I applied (female). The position was given to a maleâĂęwho made the argument that I was better suited to continue doing science since I was the better scientist. I thought, OK; I'll focus on my science...I never received much financial support for those efforts, but the men taking on leadership positions were provided generous resources and accolades for work done by me for free. Years pass and the center is still all men except me...Passive-aggressive and overt back-stabbing is common in my direction. Campus leadership tends to appoint the same pool of men to leadership positions...Power is concentrated amongst a too-small pool of men...I need to be surrounded by women in power that have my back. The men here on campus are not on board for that. I will retire next year...I am tired. - white tenured woman, medicine

*I'm non-binary, I don't fit, and I didn't from the beginning. There are so many constructs in place that [administrators] can legitimately useâĂęto get rid of me. For example, [student evaluations] have been thoroughly investigated in peer-reviewed research as discriminating against [marginalized] instructors. I provide actual *evidence* of student learning, but I can't do any better than "meets expectations" because of those [student evaluations]. My research is continually dismissed. I'm tired of being told that I have no value...I worked for many years to get here and now I can't wait to get out.* - white pre-tenure non-binary faculty member in STEM

B. Particular salience of devaluation for women of color

I'm currently considering leaving the position (full prof). At this point in my career, the frustration has reached the point where I don't enjoy the science any longer. The main and only reason for leaving has been a lack of support and discriminatory treatment along my whole career as a professor. When my productivity was high (bringing in money, publishing, graduating students) those things were not valued. When I needed infrastructure to maintain my laboratory I was left waiting ([over a decade] to fix lab issues)...When I complained I was told that my complaint was not valid, when I went [up] for promotion I was told the standard had to be higher, and then the next person (male) in line was promoted with less accomplishments. When I published in [in the most prestigious journals] I was told the important thing was the funding, when I brought in funding I was told I had to publish more. I created new courses, received several teaching awards, and have done more service than most of my colleagues, but my salary is still lower than most of my colleagues...I still enjoy teaching and that is the reason I haven't quit yet, but most likely this will happen at the end of this academic year. - Hispanic tenured woman, chemistry

Unfortunately racism has persisted, including that against Asian Americans, which is often invisible and viewed as unimportant and thus not taken seriously. For me this has meant emotional and support work for Asian American students, as well as calling out subtle racism even in [department] and college meetings by faculty and administrators. This is tiring and makes me want to leave. - Asian tenured woman, sociology

I have seen and experienced the mistreatment of my peers and students. And I have been marginalized because of my intersecting identities and because I speak out. Recently I have learned to just stay quiet and try to avoid meetings. It is only through this kind of disengagement that I will be able to make it to the minimum retirement age. The discrimination and toxic school and departmental environment have just done too much damage. I wish I would have left years earlier even though it would have had a negative impact on my family. But staying has had a significant and negative impact on me. - tenured woman of color, nursing

Table **S11**: **Devaluation and retention.** Exemplar quotations from women and non-binary faculty who reported that (A) they were considering leaving due to the combination of devaluation and a lack of institutional response, which (B) was particularly salient for women of color.

A. Procedural fairness in formal evaluations

The tenure process needs to be more transparent...I've seen several junior male faculty be rewarded for schmoozing and rubbing shoulders with higher-up male faculty in ways that are unavailable to me. I would prefer the entire academic culture be re-focused on actual productivity, rather than "who you know." - white pre-tenure woman, engineering

I feel undervalued in multiple dimensions of my work...I am paid significantly less than junior male colleagues who are less productive in publications, grants and have lower teaching evaluations, but a raise will not be considered without external offers. - white tenured woman, anthropology

As a Black woman (single with no children), I still have expectations in my extended family and community to "contribute" and to be a caregiver. These forms of caregiving are often invisible, including mentoring and emotional support for students of color who are not my "official" graduate advisees or students. Hence, I think the field is exceptionally demanding and tough, but falls more heavily on women and women of color given structural inequities and invisible forms of labor that we carry. What needs to be different are equity and retention initiatives that face these issues head-on, in terms of intentionally disrupting normative and structural inequities in the field - this could look like offering more course releases, more grant opportunities, or more sabbaticals to women, faculty of color, and single parents. I have recently earned tenure and will have a sabbatical, but it feels like I owe much of my outcome to a combination of luck, extreme and unhealthy sacrifices to my health (mental and physical), as well as wise mentoring and a dean that intervened to offer resources when I was truly down and approaching burnout. - Black tenured woman, education

B. Creating collegial environments

It wasn't intentional, but I can't tell you how many people I had to remind to please not ask things of me while I was on maternity leave. One admin wouldn't take no as an answer and didn't understand how emailing asking for signatures wasn't "that big of deal for me to do" during my leave. I even had the chair ask me to teach for him multiple times while I was on leave - how do you say no to your boss? I had a rough pregnancy with an additional surgery, so I couldn't walk at that time, let alone teach. Our department still routinely has meetings after 5pm - and folks seem a bit clueless when I try to remind them that some of us have kiddos we want to see. - white tenured mother, engineering

There needs to be more diversity in the full time faculty poolâĂęUntil [recently], approx 15% were women. Now, we are at 25% after raising the issue numerous times...[less than 10%] identify as women [of color]. These numbers are dismal. I face microaggressions almost every day, and I am one of the lucky ones. I have a lot of support from my colleagues and my department, but entrenched racism cannot be undone overnight. There is still a "boys club" that makes decisions without involving women and then presents them as the default. - Asian tenured woman, computer science

C. Misconduct reporting structures

As a whistle-blower, who was retaliated against, but managed to survive years of being effectively black-listed, my number one solution is a commitment to addressing misconduct fairly and efficiently, in a manner that protects the reporter. Systemic discrimination CAN NOT BE ADDRESSED without a commitment to punishing misconduct. No amount of online trainings will change the behavior of people who feel entitled to commit misconduct. Reporting structures need to be transparent and easy to access. Someone who reports significant misconduct...needs to be assigned an advocate...who will help to monitor what happens after they report. Misconduct reporting structures need to involve possible paths outside of the reporter's chain of command. Reporters need options (anonymous, confidential, anonymous and confidential, etc.) for how the information will be handled and clear guidance about who will receive information they report. Misconduct data should be collected, aggregated, and reportedâĂęHow many reports of various types of misconduct occurred and what was the outcome? What were the penalties for perpetrators? Misconduct records should be auditable by relevant agencies (e.g., funding agencies). Even if someone reports something and asks that no action be taken, if there is a record of a report, and the perpetrator does it again to someone else, then the institution might be able to do something about it. No amount of yoga, meditation, and chamomile tea will fix the problems that I am talking about! The over-emphasis on "positive thinking" by institutions is basically gaslighting to deflect attention from the institution's own failings and unwillingness to address difficult problems. Anyone who has been harassed or discriminated against knows what a load of BS a lot of the "wellness" programming is. - white woman

Stop with the incessant DEI programs that are not helping. Instead implement useful programs...Be able to punish and fire harassers and bullies, even if they have tenure. Leadership [needs to] have the guts to do the right thing. - white tenured woman, physics

Table **S12**: **Interventions.** Exemplar quotations from women faculty about what could be done to address devaluation. They recommended interventions that (A) enforce procedural fairness in formal evaluation processes, (B) create collegial, inclusive environments free of subtle exclusionary behaviors, and (C) improve misconduct reporting structures.

S3. Quantitative design and analysis

A. Regression: Reporting each topic by group

We were interested in comparing the frequency counts of topics reported by four groups: white women, women of color, white men, and men of color (Fig. 2B). In order to quantify which group of faculty was most likely to report a given topic T, we fit a separate logistic regression model to the dataset to model the probably t of reporting each topic T, adjusting for each group (white women, women of color, white men, and men of color), group type (whether they are current versus former faculty), career age (years since PhD, ranging from 1 to 60), discipline (one of 29), and faculty employer prestige (scaled such that a 1 unit increase corresponds to a 1 decile increase up the prestige hierarchy, but note that each rank in the regression represents a unique institution; Section S1A):

$$logit(t) = \beta_0 + \beta_1[\text{Women of Color (WOC)}] + \beta_2[\text{White Women (WW)}] + \beta_3[\text{Men of Color (MOC)}] + \beta_4[\text{Group type}] + \beta_5[\text{Career age}] + \beta_5[\text{Prestige decile}] + \beta_F[\text{Discipline}]$$

where F represents 29 different coefficients, one for each discipline.

For the groups, rather than using standard coding that compares each group to a reference group (e.g., dropping white men and comparing all other groups to them), we use sum-to-zero coding, a contrast coding scheme that compares each level of a categorical variable to the overall mean of the dependent variable across all levels. This allows us to compare the odds of one group reporting a topic to the odds of the average faculty respondent reporting that topic. The coefficients for White Men (WM) are computed by taking the negative of the sum of the other coefficients.

Topic	$\hat{\boldsymbol{\beta}}_{WOC}$	$\hat{\boldsymbol{\beta}}_{WW}$	$\hat{\boldsymbol{\beta}}_{MOC}$	$\hat{\boldsymbol{\beta}}_{WM}$
Research	−0.26	0.06	0.12	0.08
Teaching	−0.07	0.47∗∗∗	−0.35	−0.05
Service	−0.07	0.47∗∗∗	−0.35	−0.05
Caretaking	0.66∗∗∗	0.78∗∗∗	−0.88∗∗	−0.56∗∗∗
Workload	0.20	0.51∗∗∗	−0.67∗∗∗	−0.04
Location	0.14	0.17	−0.15	−0.16
Leadership	0.12	0.05	−0.23∗	0.06
Resources	−0.08	0.36∗∗∗	−0.26	−0.01
Admin. policies	0.33∗	0.46∗∗∗	−0.48∗	−0.31∗∗
Environment	0.27∗	0.02	−0.03	−0.26∗∗∗
Ineq. work	0.67∗∗∗	0.55∗∗∗	−0.31	−0.91∗∗∗
Devaluation	0.83∗∗∗	0.32∗∗∗	−0.24∗	−0.90∗∗∗

* $p < 0.05$; ** $p < 0.01$; *** $p < 0.001$.

Table **S13**: Coefficients for each group reporting each topic by group compared to the mean, adjusting for whether they are current or former faculty, career age, discipline, and prestige decile

B. Regression: Reporting devaluation

In order to more easily assess the relative importance of various covariates on feeling devalued, we fit another logistic regression to model their probability p of reporting the topic of devaluation (1 = reported devaluation, 0 = did not report devaluation), using faculty gender (0 = man, 1 = woman), race (0 = white, 1 = person of color), rank (0 = pre-tenure, 1 = tenured), whether they are in a STEM or non-STEM domain (0 = non-STEM, 1 = STEM), whether they were employed by a high-prestige institution (0 = bottom half of the hierarchy, 1 = top half of the hierarchy), and whether they were current faculty or not (0 = former, 1 = current):

$$logit(p) = \beta_0 + \beta_1[\text{Women}] + \beta_2[\text{BIPOC}] + \beta_3[\text{Tenured}] + \beta_4[\text{STEM}] + \beta_5[\text{High prestige}] + \beta_6[\text{Current faculty}]$$

If the likelihood of reporting devaluation does not depend on gender, the odds that a woman reports devaluation ($odds_W$) should be equal to the odds that a man reports devaluation ($odds_M$), so $odds_W/odds_M$ (the odds ratio), which is equal to $\exp(\beta_1)$, should be 1. An odds ratio that is less than 1 means that men are more likely to report devaluation than women, and an odds ratio that is greater than 1 means that women are more likely to report devaluation than men (**Table S14**).

Devaluation	$\hat{\boldsymbol{\beta}}$	$\exp(\hat{\boldsymbol{\beta}})$	95% CI	z
Women	1.21***	3.36	(2.95, 3.83)	18.2
BIPOC	0.57***	1.77	(1.51, 2.08)	7.0
Tenured	−0.47***	0.63	(0.52, 0.75)	−5.1
STEM	−0.09	0.91	(0.80, 1.04)	−1.4
High prestige	0.02	1.02	(0.88, 1.18)	0.2
Current faculty	0.10	1.10	(0.96, 1.27)	1.3

* $p < 0.05$; ** $p < 0.01$; *** $p < 0.001$.

Table **S14**: Odds of reporting devaluation, adjusting for gender, race, whether in a STEM domain or not, whether employed at a high-prestige institution or not (top half of the hierarchy), and whether current faculty (vs former faculty).

We also calculated the odds of reporting devaluation by each domain of study. However, instead of using binned covariates (e.g., STEM vs non-STEM, tenured vs pre-tenure, and high-prestige versus low) we used the more detailed versions. Using these more detailed versions only slightly reduced the coefficients (**Table S15**). Gendered odds of reporting devaluation for each domain of study are in **Table S16**.

Devaluation	$\hat{\boldsymbol{\beta}}$	$\exp(\hat{\boldsymbol{\beta}})$	95% CI	z
Women	1.11***	3.03	(2.63, 3.50)	15.2
BIPOC	0.46***	1.59	(1.34, 1.89)	5.3
Career age	−0.03***	0.97	(0.96, 0.98)	−8.9
Prestige decile	0.01	1.10	(0.98, 1.04)	0.8
Current faculty	−0.07	0.92	(0.79, 1.08)	−1.0

* $p < 0.05$; ** $p < 0.01$; *** $p < 0.001$.

Table **S15**: Odds of reporting devaluation, adjusting for gender, race, domain of study (not shown), institutional prestige, and whether current faculty (vs former faculty).

Field	$\hat{\beta}$	$\exp(\hat{\beta})$	95% CI	z
**Academia**	1.24***	3.45	(3.09, 3.89)	19.1
**STEM**	1.33***	3.76	(3.05, 4.48)	13.7
Natural Sciences	1.28***	3.59	(2.70, 4.48)	10.2
Engineering	1.79***	5.98	(2.64, 9.32)	6.6
Formal Sciences	1.12***	3.05	(1.74, 4.36)	5.3
Medicine and Health	1.28***	3.61	(1.64, 5.57)	4.8
**Non-STEM**	1.12***	3.06	(2.51, 3.61)	12.3
Humanities	1.07***	2.92	(1.84, 4.01)	5.8
Social Sciences	1.30***	3.67	(2.30, 5.03)	7.0
Applied Sciences	1.11***	3.03	(1.63, 4.43)	4.9
Education	1.26	3.54	(−0.88, 7.96)	2.4

* $p < 0.05$; ** $p < 0.01$; *** $p < 0.001$.

Table **S16**: Gendered odds of reporting devaluation across domains of study, adjusting for gender, race, career age, discipline, prestige decile, and whether current or former faculty

C. Conditional probabilities and mediation analysis

In order to understand how each topic T might independently relate to considering leaving a job, we computed conditional probabilities for all 12 topics among current faculty:

$$P(\text{reported topic } T|\text{considers leaving}) = \frac{P(\text{reported topic } T \text{ AND considers leaving})}{P(\text{considers leaving})}$$

We marked current faculty as *considering leaving* if they selected leaving for positive and/or negative reasons in the survey (Section S1B). They were only marked as *not considering leaving* if they explicitly selected "I would not consider leaving."

We computed probabilities separately among men and women current faculty (Fig. 5A).

Mediation. While these probabilities were calculated independently, a multiple mediation model allows us to examine which topics best explain the relationship between gender and retention while accounting for their potential interrelationships as well as other potentially influential covariates [12]. We fit two separate models for current versus former faculty. In each of the models, the retention outcome variable was defined differently. The model for current faculty estimated the effect of gender on considering leaving a position, while the model for former faculty estimated the effect of gender on actually leaving a position (and included current faculty as the group who did not leave). We included all 12 topics as well as faculty race, whether they were in a STEM field, institutional prestige, and whether they were tenured in the models.

The first step of a mediation analysis is to define the paths from gender to each of the mediators:

```
is_deval ~ a1 * is_female
is_research ~ a2 * is_female
is_teaching ~ a3 * is_female
is_service ~ a4 * is_female
is_caring ~ a5 * is_female
is_workload ~ a6 * is_female
is_location ~ a7 * is_female
is_leadership ~ a8 * is_female
is_resources ~ a9 * is_female
is_policies ~ a10 * is_female
is_environment ~ a11 * is_female
is_ineq ~ a12 * is_female

is_STEM ~ a13 * is_female
prestige_decile ~ a14 * is_female
is_POC ~ a15 * is_female
is_tenured ~ a16 * is_female
```

Next, we define the paths from the mediators to the retention outcome variable.

```
# model 1: current faculty
is_considering_leaving ~ b1 * is_deval + b2 * is_research + b3 * is_teaching +
                         b4 * is_service + b5 * is_caring + b6 * is_workload +
                         b7 * is_location + b8 * is_leadership + b9 * is_resources +
                         b10 * is_policies + b11 * is_environment + b12 * is_ineq +
                         b13 * is_STEM + b14 * prestige_decile + b15 * is_POC +
                         b16 * is_tenured

# model 2: all faculty
left ~ b1 * is_deval + b2 * is_research + b3 * is_teaching +
       b4 * is_service + b5 * is_caring + b6 * is_workload +
       b7 * is_location + b8 * is_leadership + b9 * is_resources +
       b10 * is_policies + b11 * is_environment + b12 * is_ineq +
```

```
        b13 * is_STEM + b14 * prestige_decile + b15 * is_POC +
        b16 * is_tenured
```

Finally, we define the direct effect of gender on the retention variable.

```
# model 1: current faculty
is_considering_leaving ~ c * is_female

# model 2: all faculty
left ~ c * is_female
```

After defining the model, we initialize and fit it with default parameters from the `semopy` package [13] to get estimates for `a1-a16` and `b1-b16`, as well as the direct effect `c_prime`. We then compute each indirect effect as follows:

```
indirect_deval = a1 * b1
indirect_research = a2 * b2
indirect_teaching = a3 * b3
indirect_service = a4 * b4
indirect_caring = a5 * b5
indirect_workload = a6 * b6
indirect_location = a7 * b7
indirect_leadership = a8 * b8
indirect_resources = a9 * b9
indirect_policies = a10 * b10
indirect_environment = a11 * b11
indirect_ineq = a12 * b12

indirect_STEM = a13 * b13
indirect_prestige = a14 * b14
indirect_race = a15 * b15
indirect_tenured = a166 * b16
```

Finally, we compute the total effect as:

```
total_effect = cprime +
               indirect_deval +
               indirect_research + indirect_teaching + indirect_service +
               indirect_caring + indirect_workload + indirect_location +
               indirect_leadership + indirect_resources + indirect_policies +
               indirect_environment + indirect_ineq +
               indirect_STEM + indirect_prestige + indirect_race + indirect_tenured
```

Estimates for model 1 are in **Table S17** and estimates for model 2 are in **Table S18**.

Operation	Variable	Estimate	z
is_deval ~ is_female	a1	0.47***	17.6
is_research ~ is_female	a2	−0.03*	−2.5
is_teaching ~ is_female	a3	0.04***	4.5
is_service ~ is_female	a4	0.04***	4.5
is_caring ~ is_female	a5	0.09***	11.5
is_workload ~ is_female	a6	0.09***	7.1
is_location ~ is_female	a7	0.01**	3.0
is_leadership ~ is_female	a8	0.03	1.9
is_resources ~ is_female	a9	0.05***	3.9
is_policies ~ is_female	a10	0.06***	7.5
is_environment ~ is_female	a11	0.07***	5.4
is_ineq ~ is_female	a12	0.09***	11.3
is_STEM ~ is_female	a13	−0.23***	−14.3
prestige_decile ~ is_female	a14	−0.07	−0.9
is_POC ~ is_female	a15	0.04**	3.1
is_tenured ~ is_female	a16	−0.05***	−5.1
is_considering_leaving ~ is_deval	b1	0.15***	18.9
is_considering_leaving ~ is_research	b2	0.06***	3.9
is_considering_leaving ~ is_teaching	b3	0.01	0.7
is_considering_leaving ~ is_service	b4	0.01	0.7
is_considering_leaving ~ is_caring	b5	−0.01	−0.7
is_considering_leaving ~ is_workload	b6	0.07***	4.0
is_considering_leaving ~ is_location	b7	0.09*	2.2
is_considering_leaving ~ is_leadership	b8	0.11***	7.6
is_considering_leaving ~ is_resources	b9	0.04*	2.2
is_considering_leaving ~ is_policies	b10	0.00	0.0
is_considering_leaving ~ is_environment	b11	−0.01	−0.3
is_considering_leaving ~ is_ineq	b12	0.06*	2.1
is_considering_leaving ~ is_STEM	b13	0.02	1.8
is_considering_leaving ~ prestige_decile	b14	−0.00	−1.8
is_considering_leaving ~ is_POC	b15	−0.04*	−2.5
is_considering_leaving ~ is_tenured	b16	−0.09***	−4.0
is_considering_leaving ~ is_female	c_prime	0.03	1.7

* $p < 0.05$; ** $p < 0.01$; *** $p < 0.001$.

Table **S17**: Mediation estimates for considering leaving a job, among current faculty.

Operation	Variable	Estimate	z
`is_deval ~ is_female`	a1	0.59***	13.1
`is_research ~ is_female`	a2	0.01	0.6
`is_teaching ~ is_female`	a3	−0.00	−0.1
`is_service ~ is_female`	a4	−0.00	−0.1
`is_caring ~ is_female`	a5	0.05***	5.7
`is_workload ~ is_female`	a6	0.06***	4.3
`is_location ~ is_female`	a7	0.04*	2.1
`is_leadership ~ is_female`	a8	0.09***	4.0
`is_resources ~ is_female`	a9	0.03*	2.4
`is_policies ~ is_female`	a10	0.09***	6.0
`is_environment ~ is_female`	a11	0.06***	3.5
`is_ineq ~ is_female`	a12	0.05***	5.9
`is_STEM ~ is_female`	a13	−0.26***	−10.7
`prestige_decile ~ is_female`	a14	0.04	0.3
`is_POC ~ is_female`	a15	0.03	1.8
`is_tenured ~ is_female`	a16	−0.13***	−6.1
`left ~ is_deval`	b1	0.16***	6.6
`left ~ is_research`	b2	0.11**	3.1
`left ~ is_teaching`	b3	0.01	0.3
`left ~ is_service`	b4	0.01	0.3
`left ~ is_caring`	b5	−0.11*	−2.0
`left ~ is_workload`	b6	0.03	0.8
`left ~ is_location`	b7	0.08**	3.0
`left ~ is_leadership`	b8	0.19***	8.7
`left ~ is_resources`	b9	0.12***	3.6
`left ~ is_policies`	b10	0.10**	2.9
`left ~ is_environment`	b11	0.13***	4.5
`left ~ is_ineq`	b12	0.06	1.0
`left ~ is_STEM`	b13	−0.00	−0.3
`left ~ prestige_decile`	b14	−0.00	−0.7
`left ~ is_POC`	b15	0.04	1.6
`left ~ is_tenured`	b16	−0.13***	−5.3
`left ~ is_female`	c_prime	0.01	0.2

* $p < 0.05$; ** $p < 0.01$; *** $p < 0.001$.

Table **S18**: Mediation estimates for actually leaving a job, among former faculty.

S4. Supplementary analyses

A. Current faculty

In the main text, we aggregated results for current and former faculty because they were very similar, in order to reduce the complexity of presentation of results. **Fig. S2** shows the gendered frequency counts of topics reported only among current faculty (Fig. S2A) and the odds of each group of current faculty (women of color, white women, men of color, and white men) reporting each topic compared to the average current faculty member (Fig. S2B).

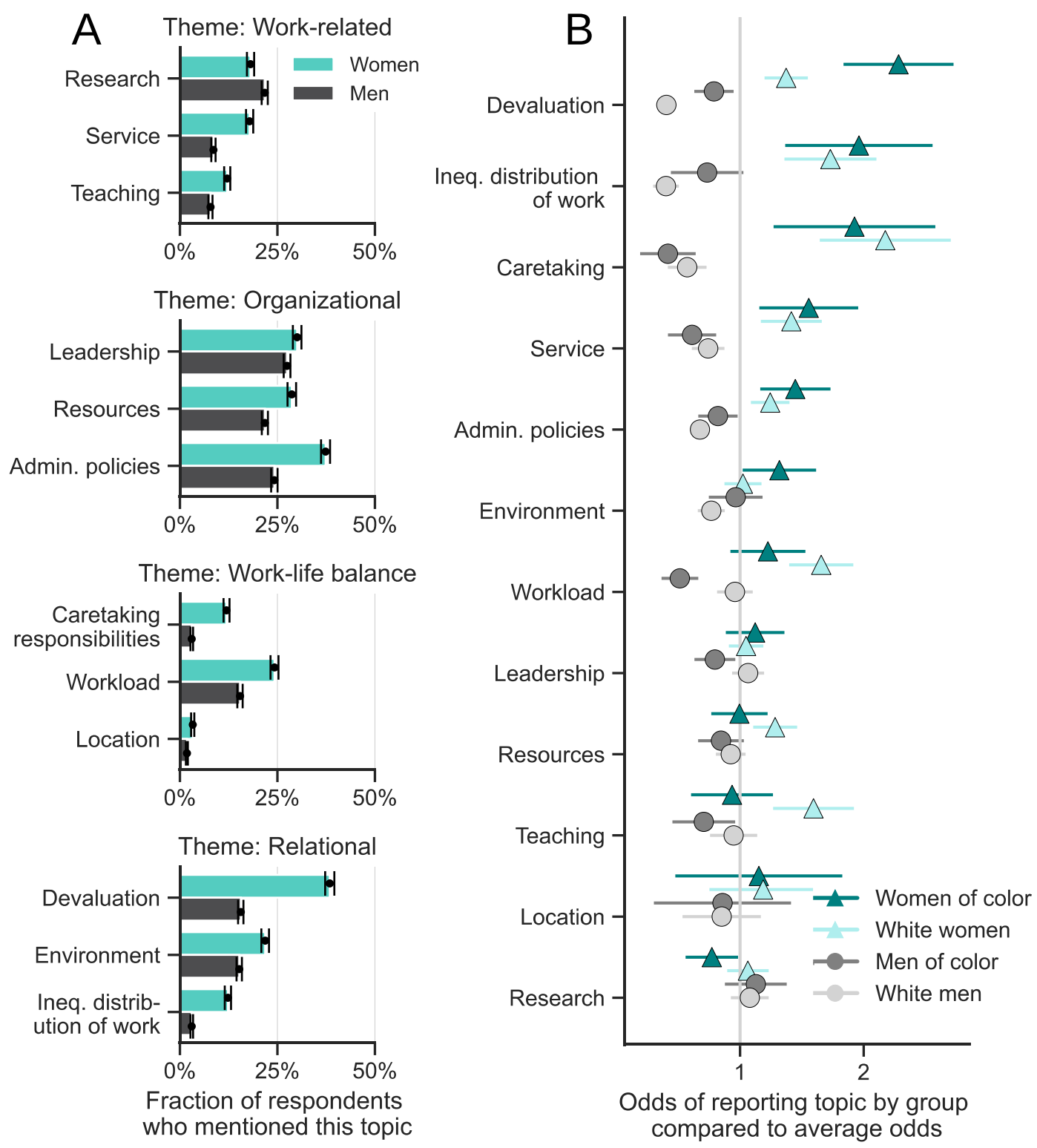

Figure **S2**: **(A)** Frequency counts of topics described by current faculty ($N = 4,172$; 168 responses excluded, see SI Sections S1B), split by self-reported binary gender. All gender differences are statistically significant at $\alpha = 0.05$ via two-proportion z tests. 95% confidence intervals shown. **(B)** Odds of each group of current faculty (women of color, white women, men of color, and white men) discussing each topic, divided by the mean odds for all current faculty, adjusting via logistic regression for career age, discipline (one of 29), and unique institutional prestige scores for each institution. 95% confidence intervals shown. An odds ratio of 1 means that group was no more likely to mention the topic than the average faculty member.

In addition to binary gender (women vs men), we also analyzed how themes varied for non-binary facuty, across race, age, parenthood, field, and institutional prestige.

We found that non-binary faculty more frequently reported devaluation than men (55% of non-binary faculty vs 16% of men; z = 4.8, N = 2,621, $p < 0.001$) and women (55% of non-binary faculty vs 38% of women; z = 1.5, N = 1,693, p = 0.13)—although the difference between non-binary faculty and women was not statistically significant, likely due to small sample sizes (**Fig. S3**). Non-binary faculty were also more likely than both women and men to mention the departmental environment (**Fig. S3**).

We also found that devaluation is the most racialized aspect of the academic workplace, with women of color most likely to report devaluation (Fig. 2B), and this pattern varies by race, with 76% of all Indigenous women reporting devaluation, compared to Hispanic women (51.3%), Black women (50.7%), and Asian women (41.3%; **Fig. S4**).

Faculty who are parents of children under the age of 18, particularly mothers, are often perceived as less competent and treated differently by their peers. However, while we find that both mothers and fathers more frequently report stress due to caretaking responsibilities and university policies such as parental leave or departmental policies such as evaluation metrics, than non-mothers and non-fathers, respectively, we find that mothers and non-mothers report devaluation at similar rates, and fathers report devaluation slightly more than non-fathers (but not nearly as much devaluation as women report; **Fig. S5**).

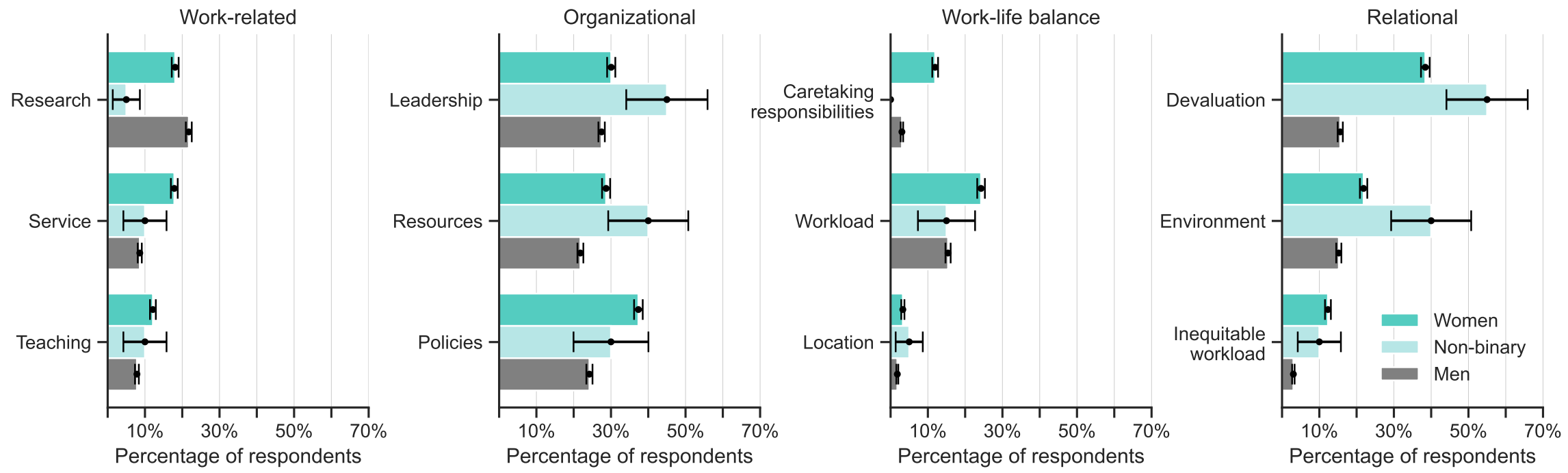

Figure **S3**: Frequency counts of topics discussed by current faculty, split by gender (women, non-binary faculty, and men). 95% confidence intervals shown.

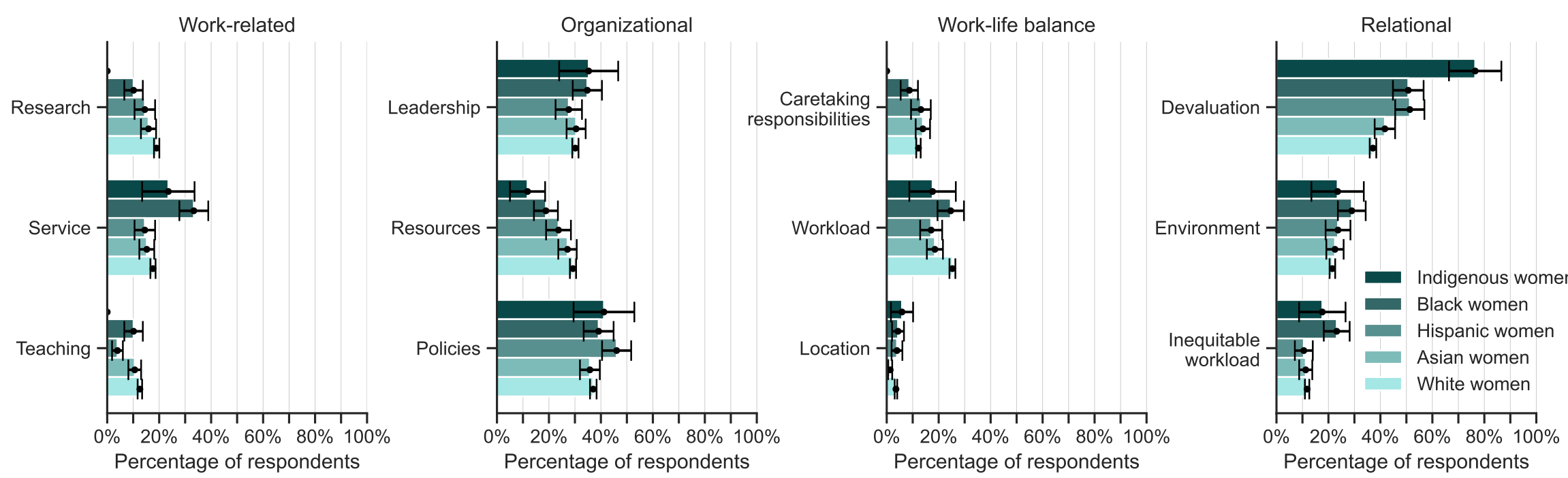

Figure **S4**: Frequency counts of topics discussed by current women faculty, split by race. 95% confidence intervals shown.

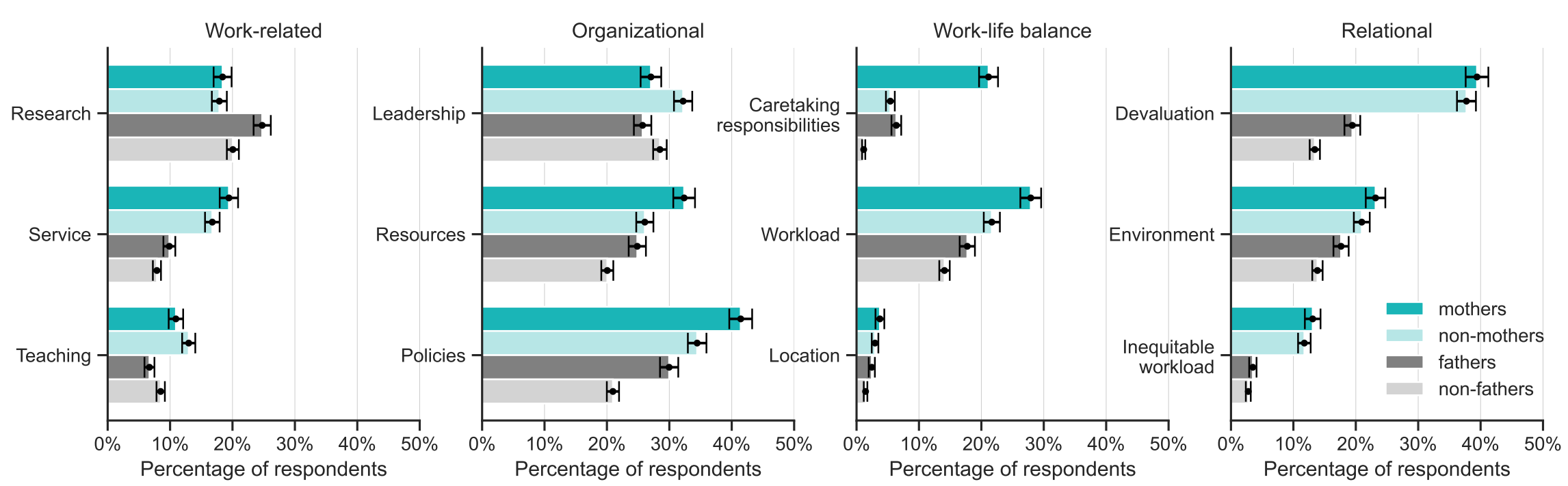

Figure **S5**: Frequency counts of topics discussed by current faculty, split by binary gender (women and men) and whether or not they are parents of kids under 18. 95% confidence intervals shown.

While it might seem intuitive that reports of feeling devalued would decrease the longer someone stays in their job, we find that current women faculty consistently report devaluation across their career, from 1 to 40+ years since they received their PhD (**Fig. S6**). In contrast, men report less devaluation later in their career compared to earlier in their career, so the gender gap in reported devaluation between men and women grows over a career, a pattern no other topic exhibits (**Fig. S6**).

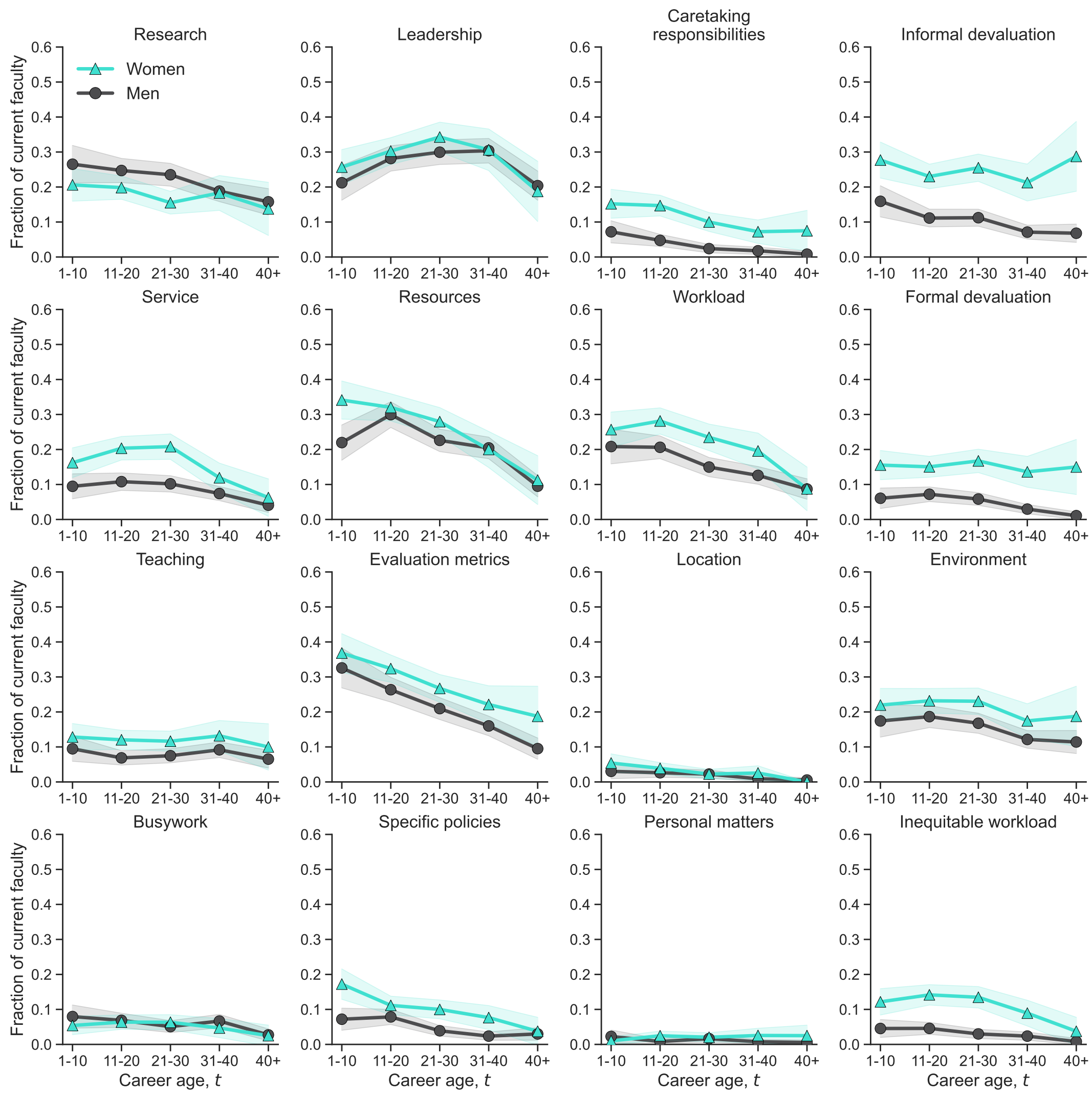

Figure **S6**: Fraction of current faculty across career age t, defined as years since a faculty member received their PhD, binned into decades, who reported each topic, split by binary gender (women and men). Envelopes indicate 95% confidence intervals.

Academic fields tend to have different norms, characteristics, values, and demographic compositions. However, we find that reports of gendered devaluation are relatively consistent across fields. Research topics such as obtaining funding and managing a research group were more frequently reported by STEM faculty (both women and men) than non-STEM faculty (**Fig. S7**). There were very few gendered differences across STEM vs non-STEM, except that non-STEM women faculty more frequently reported stress due to departmental and institutional leadership and resources (**Fig. S7**). Women in both STEM and non-STEM fields reported experiencing devaluation at similar rates (**Fig. S7**). Most individual fields followed this pattern, although some fields had larger gender gaps than others. For example, the differences between devaluation reports of women and men were 39% in engineering and 36% in economics, compared to just 15% in psychology and 18% in computer science (**Fig. S8**). This gendered variation across fields could reflect field-level norms around inclusion, competition, and collaboration.

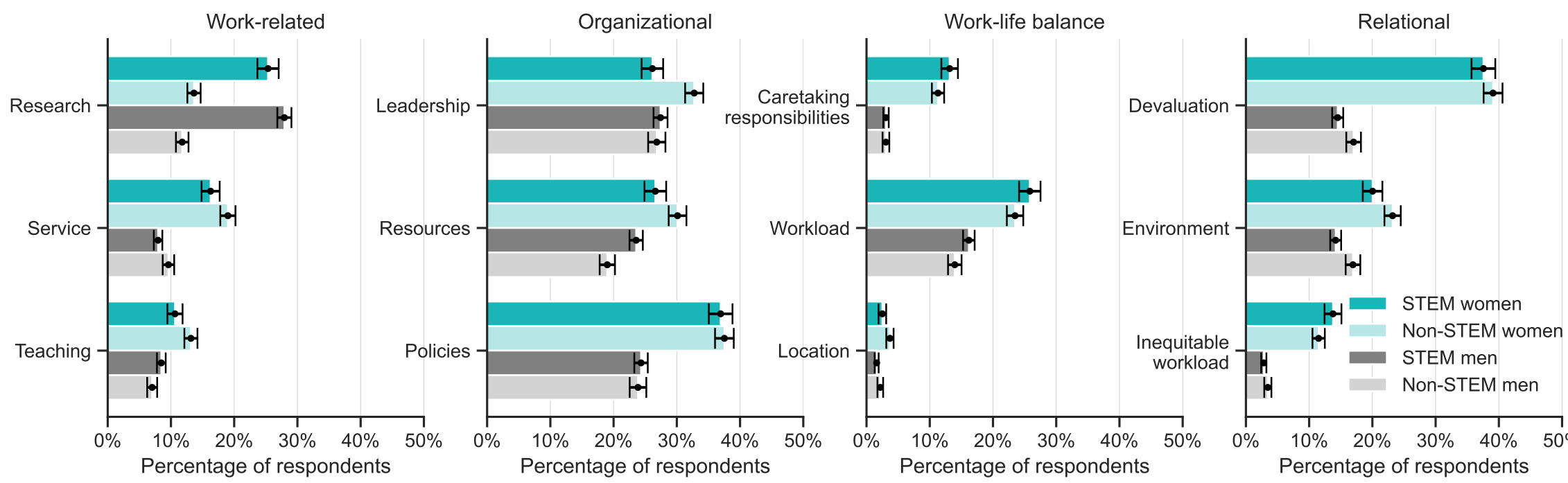

Figure **S7**: Frequency counts of topics discussed by current faculty, split by binary gender (women and men) and whether or not they are in a STEM field. 95% confidence intervals shown.

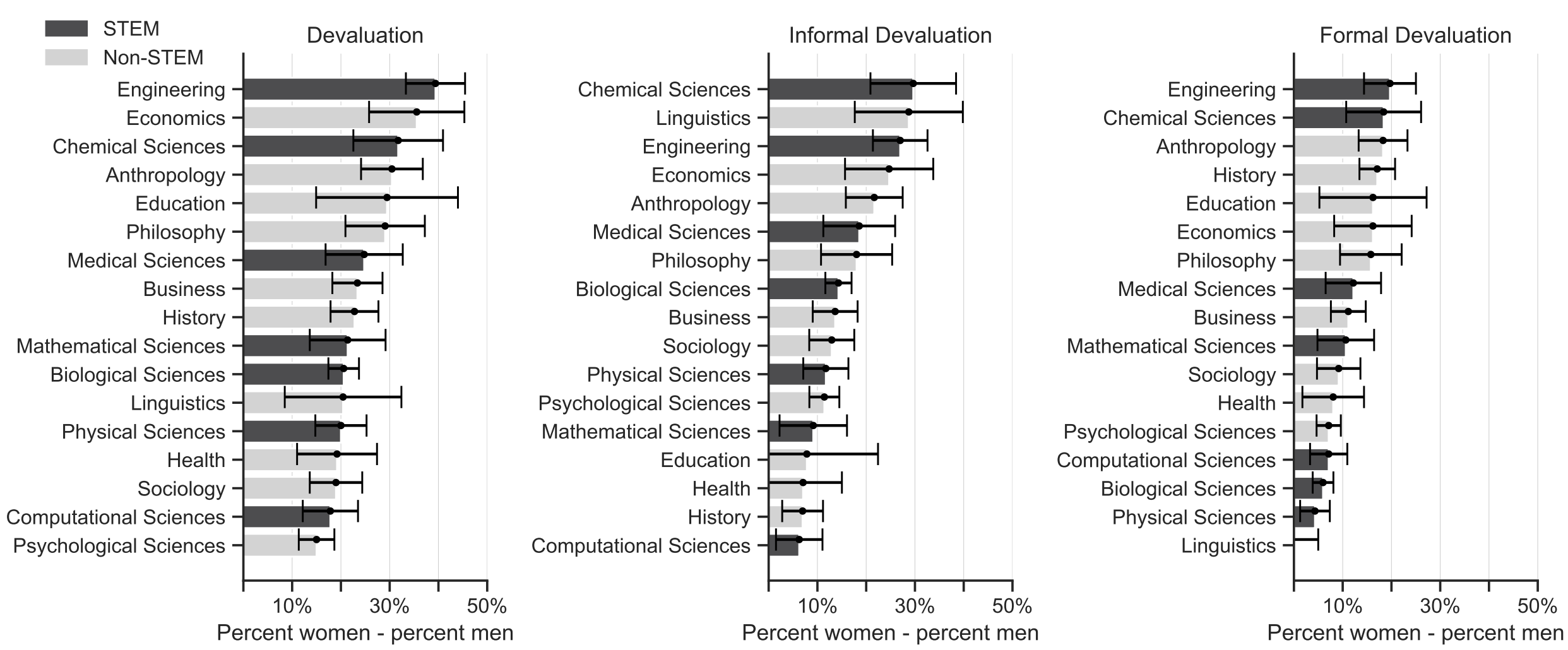

Figure **S8**: Gendered differences in reports of devaluation by academic field (the percentage of women who reported devaluation minus the percentage of men who reported devaluation), and of the tertiary themes (informal devaluation and formal devaluation). 95% confidence intervals shown.

Similarly, while a few themes varied across institutional prestige, with departmental and institutional leadership much more frequently reported by faculty at lower-prestige institutions, resource issues slightly more frequently reported by faculty at lower-prestige institutions, and research pressures slightly more frequently reported by faculty at higher-prestige institutions, none of these were particularly gendered (**Fig. S9**). In contrast, we found a large and prestige-independent gendered difference in reports of devaluation (**Fig. S9**).

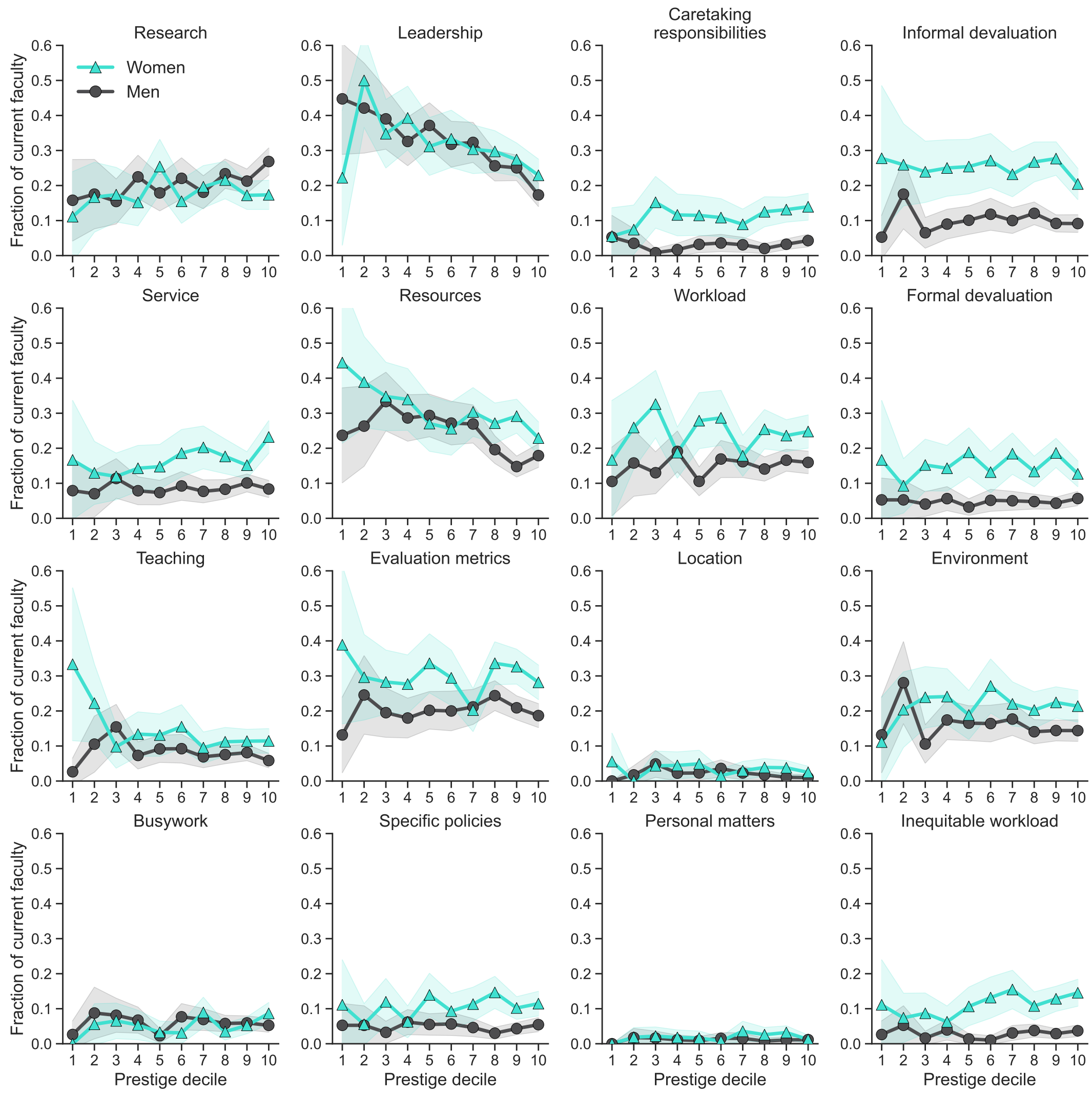

Figure **S9**: Fraction of current faculty across prestige decile (with 1 as the least prestigious decile and 10 the most prestigious) who reported each topic, split by binary gender (women and men). Envelopes indicate 95% confidence intervals.

Finally, we also examined whether the gender gap in devaluation differed by type, separating informal devaluation (e.g., social exclusion) from formal devaluation (e.g., biased evaluation). We find that women report both informal and formal devaluation at higher rates than men, with informal devaluation more common than formal devaluation across all groups. The gender gap in informal devaluation is larger than in formal devaluation, and this pattern holds across race, field, and non-binary gender identity (**Fig. S10**). Women of color report higher rates of both types than white women (**Fig. S10**), and Indigenous women report the highest rates of both informal and formal devaluation among all racial groups.

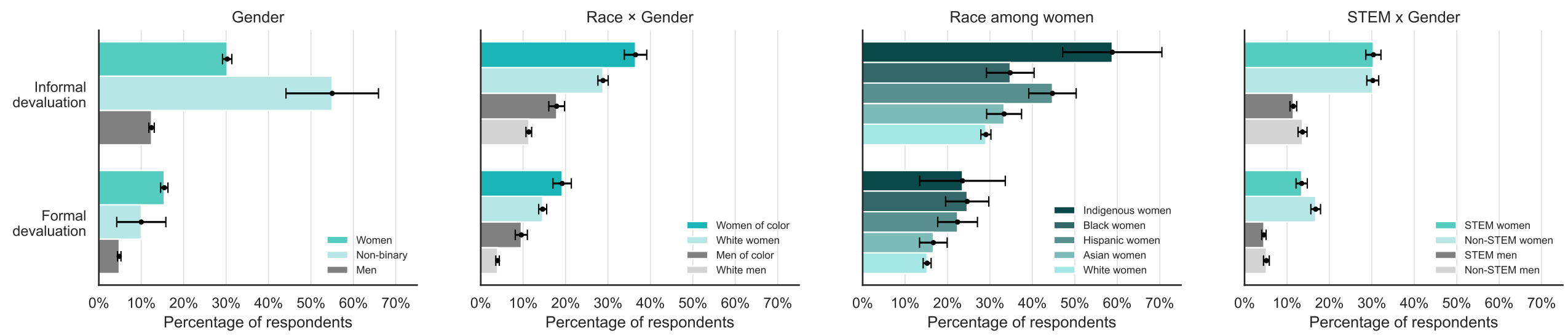

Figure **S10**: Frequency of informal and formal devaluation discussed by current faculty, split by gender (women, non-binary, and men), race and gender (women of color, white women, men of color, and white men), race among women (Indigenous, Black, Hispanic, Asian, and white women), and STEM field (STEM and non-STEM women and men). 95% confidence intervals shown.

B. Former faculty

Former faculty responses follow a similar pattern as current faculty, with devaluation as the aspect of the faculty retention with the largest gendered difference (37% of women reported devaluation vs 14% of men; **Fig. S10**). While the gender difference in reported devaluation was similar across groups, devaluation frequencies varied depending on whether faculty switched institutions, left academia, or retired. 38% of women who switched institutions reported doing so in part due to devaluation, compared to 18% of men, 48% of women who left academia reported doing so in part due to devaluation, compared to 22% of men, and 28% of women who retired reported doing so in part due to devaluation, compared to just 6% of men. Devaluation appears to be most significant for women who leave academia, and remains impactful even in women's decisions to retire.

In the main text, we aggregated results for current and former faculty because they were very similar, in order to reduce the complexity of presentation of results. **Fig. S10** shows the gendered frequency counts of topics reported only among former faculty (Fig. S10A) and the odds of each group of former faculty (women of color, white women, men of color, and white men) reporting each topic compared to the average former faculty member (Fig. S10B).

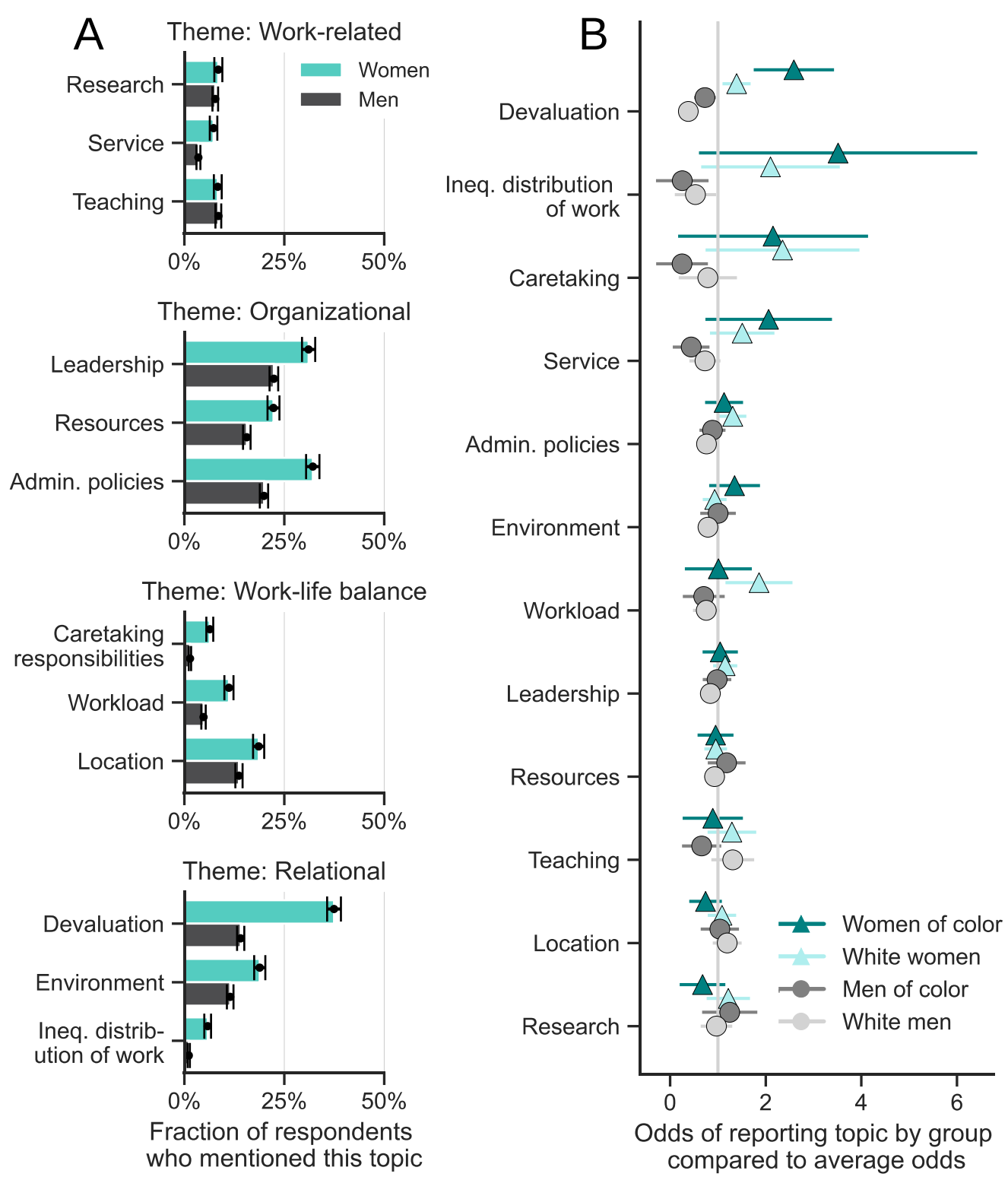

Figure **S11**: **(A)** Frequency counts of topics described by former faculty ($N = 2,197$), split by self-reported binary gender. All gender differences are statistically significant at $\alpha = 0.05$ via two-proportion z tests. 95% confidence intervals shown. **(B)** Odds of each group of current faculty (women of color, white women, men of color, and white men) discussing each topic, divided by the mean odds for all former faculty, adjusting via logistic regression for career age, discipline (one of 29), and unique institutional prestige scores for each institution. 95% confidence intervals shown. An odds ratio of 1 means that group was no more likely to mention the topic than the average faculty member.

In addition to binary gender (women vs men), we also analyzed how themes varied across race, age, parenthood, field, and institutional prestige.

Similar to current faculty, we found that devaluation is the most racialized aspect of reasons for leaving, with women of color most likely to report devaluation (Fig. 10B), and this pattern varies by race, with the majority of Indigenous women who left their jobs reporting devaluation, compared to roughly half of Hispanic and Black women, but only one third of white women (**Fig. S11**).

We find some differences among former faculty who were parents of children under the age of 18 when they left their jobs, compared to current faculty parents. While both mothers and fathers who were currently faculty more frequently report stress due to caretaking responsibilities, than non-mothers and non-fathers, respectively, we find that only mothers are more likely to report caretaking responsibilities among former faculty. In addition, mothers and non-mothers who left their jobs report devaluation at similar rates, but fathers report devaluation *significantly less often* than non-fathers (but not nearly as much devaluation as women report; **Fig. S12**).

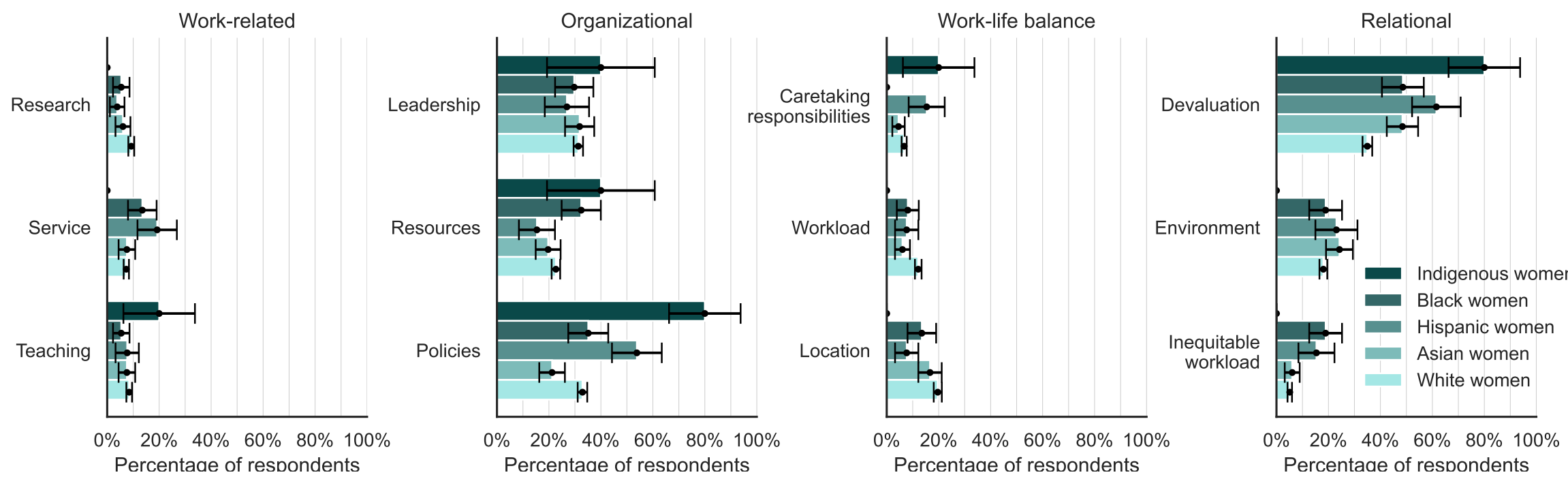

Figure **S12**: Frequency counts of topics discussed by former women faculty, split by race. 95% confidence intervals shown.

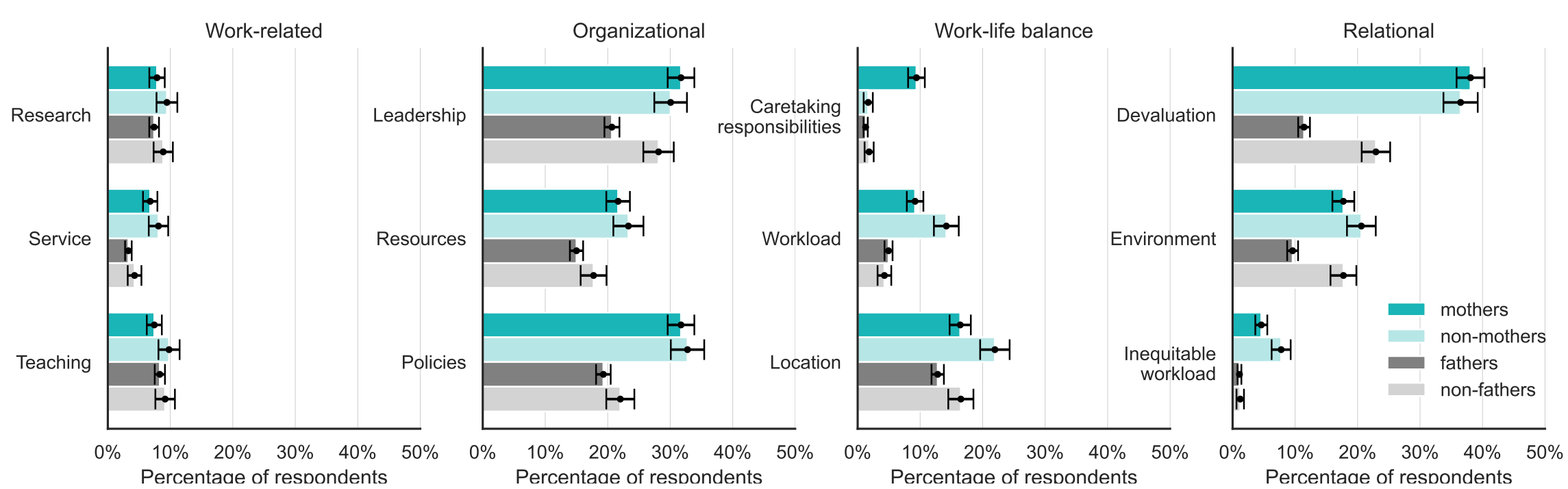

Figure **S13**: Frequency counts of topics discussed by former faculty, split by binary gender (women and men) and whether or not they were parents of kids under 18 when they left their jobs. 95% confidence intervals shown.

Similar to current women faculty, we find that former women faculty are more often to report devaluation—particularly informal devaluation—than men no matter how far into their careers they leave their jobs, from 1 to 40+ years since they received their PhD (**Fig. S13**).

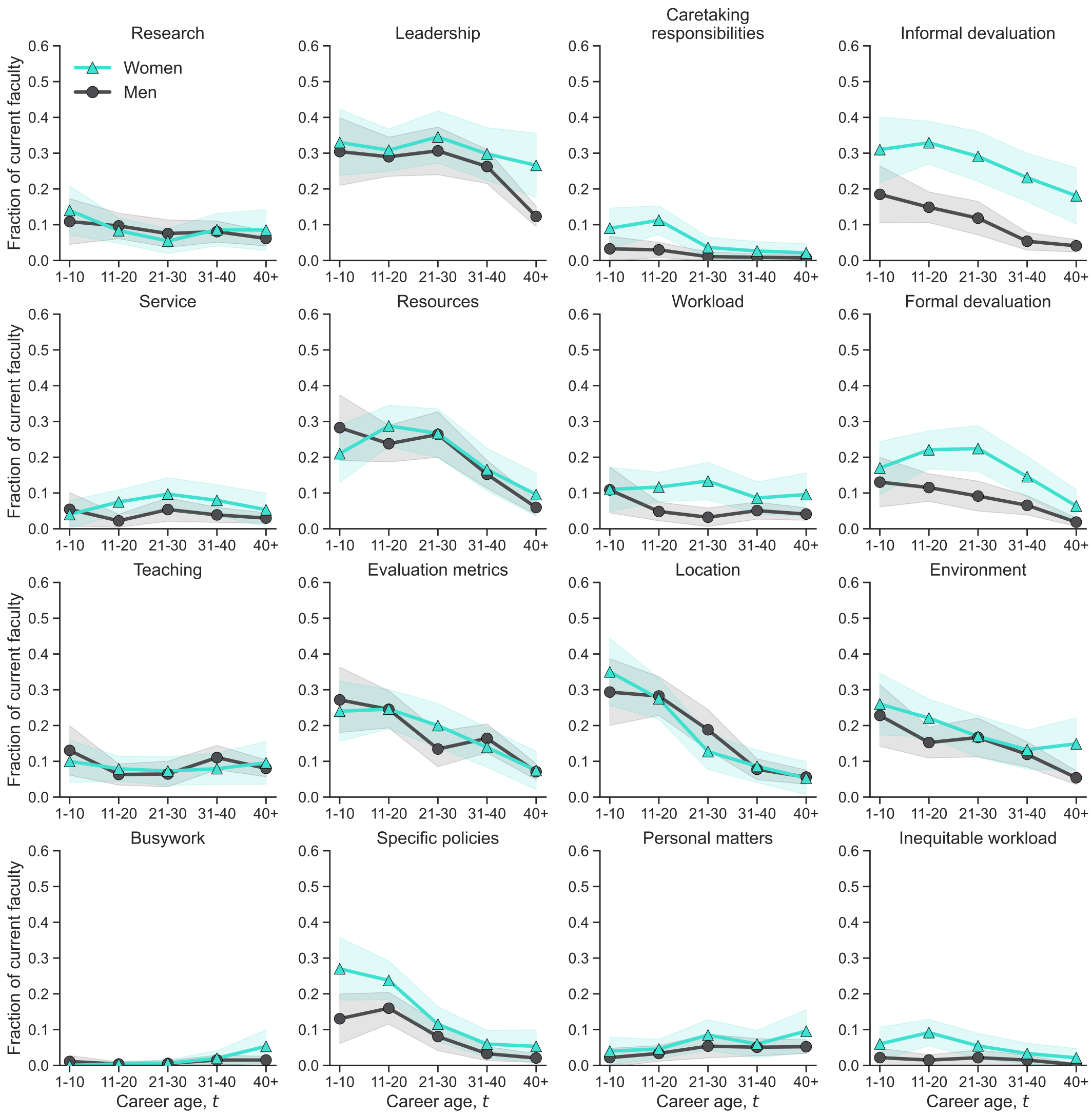

Figure **S14**: Fraction of former faculty across career age t, defined as years since a faculty member received their PhD, binned into decades, who reported each topic, split by binary gender (women and men). Envelopes indicate 95% confidence intervals.

Similar to current faculty, we find that reports of gendered devaluation among former faculty are relatively consistent across fields. Research topics such as obtaining funding and managing a research group were more frequently reported by STEM faculty (both women and men) than non-STEM faculty (**Fig. S14**). There were very few gendered differences across STEM vs non-STEM, except that non-STEM women faculty more frequently reported stress due to administrative policies (**Fig. S14**). Women in both STEM and non-STEM fields reported experiencing devaluation at similar rates (**Fig. S14**), with women in STEM slightly more often. Most individual fields followed this pattern, although some fields had larger gender gaps than others. For example, the differences between devaluation reports of women and men were highest in chemistry, medicine, biology and engineering, and lowest in economics, linguistics, philosophy, health, and physics (**Fig. S15**). This gendered variation across fields could reflect field-level norms around inclusion, competition, and collaboration.

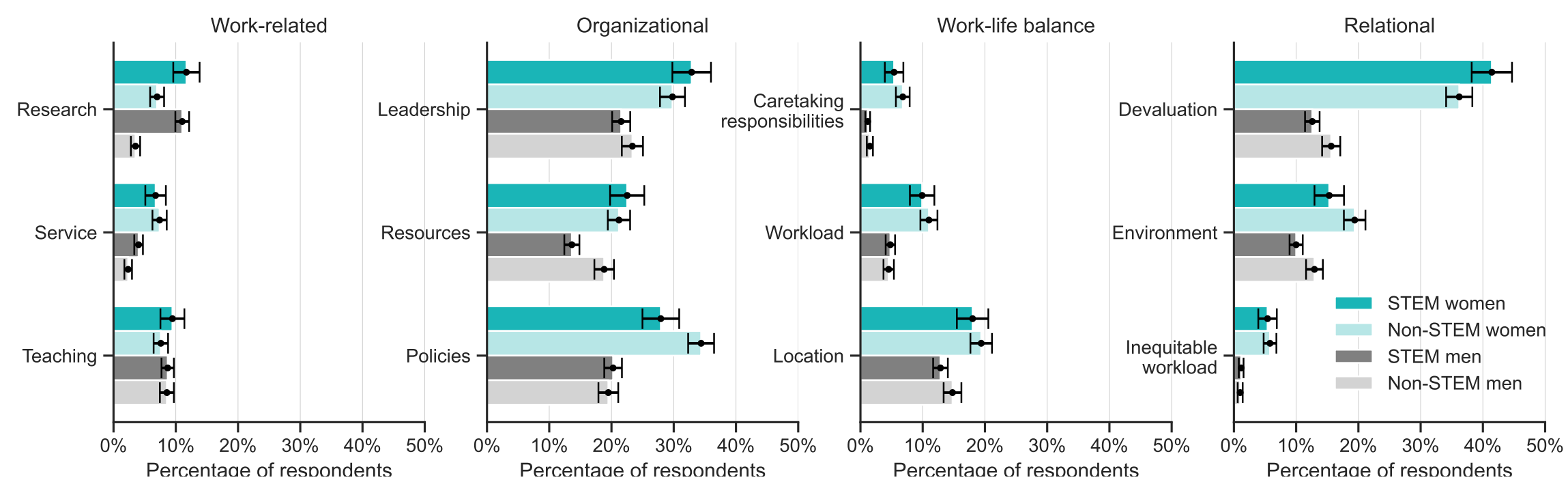

Figure **S15**: Frequency counts of topics discussed by former faculty, split by binary gender (women and men) and whether or not they are in a STEM field. 95% confidence intervals shown.

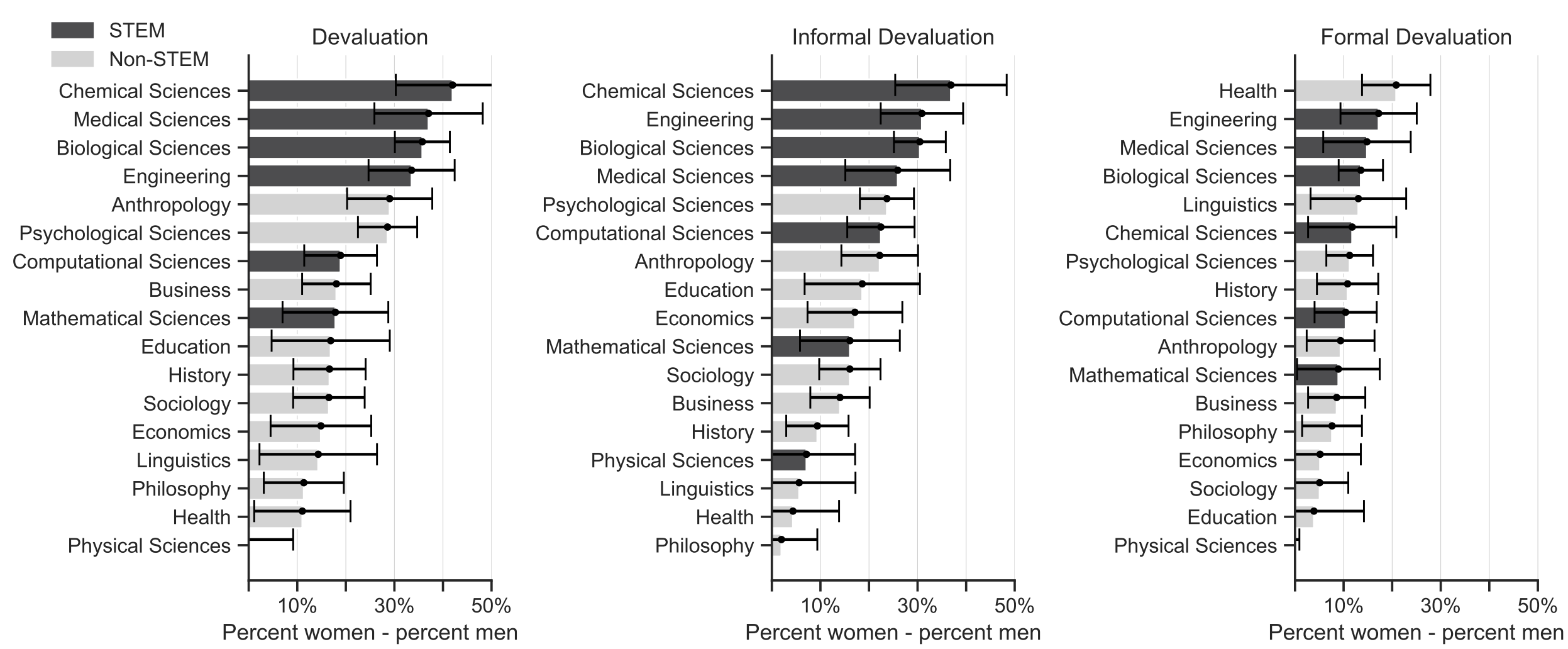

Figure **S16**: Gendered differences in reports of devaluation by academic field (the percentage of women who reported devaluation minus the percentage of men who reported devaluation), and of the tertiary themes (informal devaluation and formal devaluation). 95% confidence intervals shown.

Similarly, while a few themes varied across institutional prestige, with departmental and institutional leadership more frequently reported by faculty at lower-prestige institutions and resource issues slightly more frequently reported by faculty at lower-prestige institutions, none of these were particularly gendered (**Fig. S9**). In contrast, we found a large and mostly prestige-independent gendered difference in reports of devaluation (**Fig. S16**).

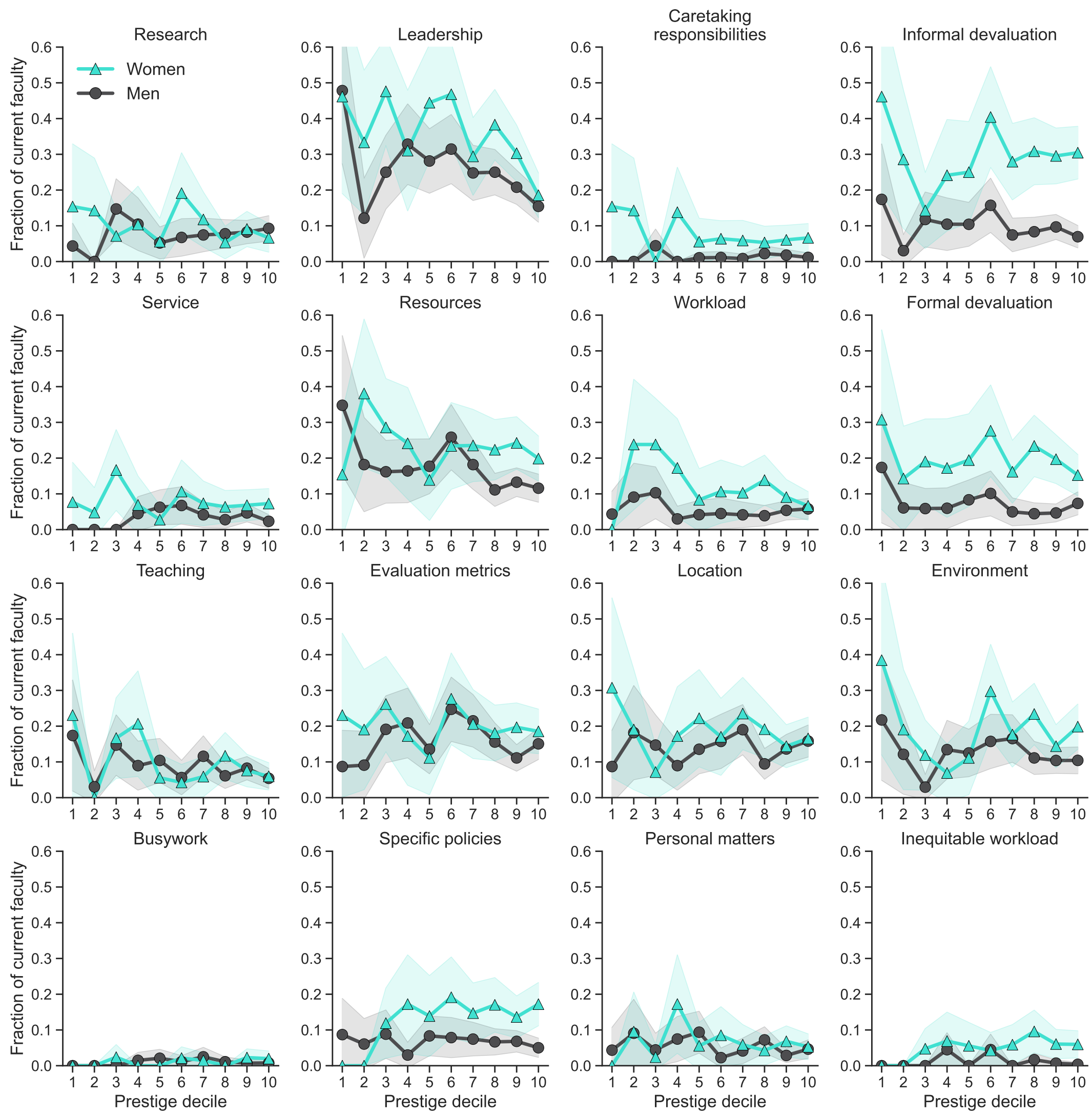

Figure **S17**: Fraction of former faculty across prestige decile (with 1 as the least prestigious decile and 10 the most prestigious) who reported each topic, split by binary gender (women and men). Envelopes indicate 95% confidence intervals.

Similar to current faculty, former women faculty report both informal and formal devaluation at higher rates than men, with informal devaluation more common than formal devaluation across all groups (**Fig. S18**). The pattern holds across race and field, with Indigenous and Black women reporting the highest rates of informal devaluation among former faculty (**Fig. S18**). The gender gap in informal devaluation is larger than in formal devaluation across all subgroups, suggesting that social exclusion and identity-based marginalization, rather than explicitly biased formal evaluation, may be the more prevalent driver of faculty attrition.

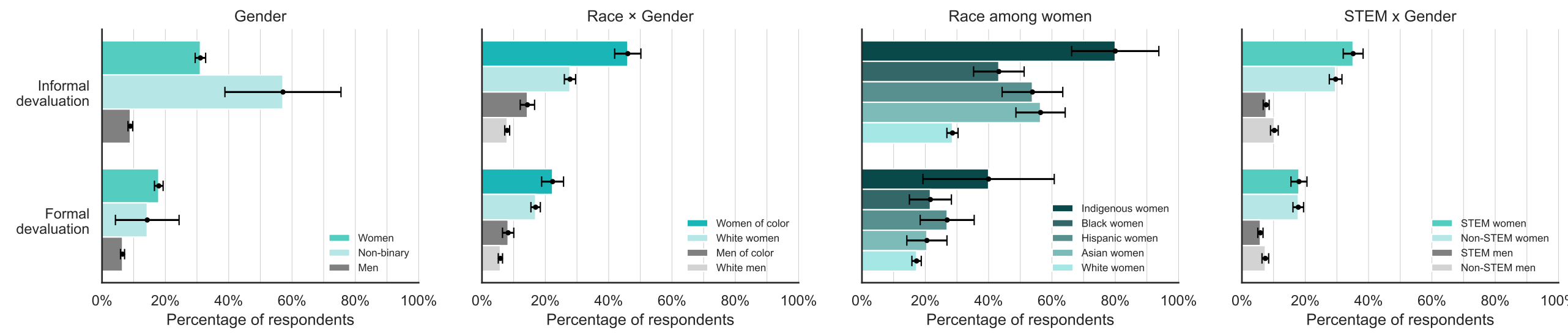

Figure **S18**: Frequency of informal and formal devaluation discussed by former faculty, split by gender (women, non-binary, and men), race and gender (women of color, white women, men of color, and white men), race among women (Indigenous, Black, Hispanic, Asian, and white women), and STEM field (STEM and non-STEM women and men). 95% confidence intervals shown.

In addition, while current women faculty who experienced formal and informal devaluation, including a lack of institutional response to the devaluation, often reported that they were considering leaving their positions, we found evidence that women do leave while analyzing the quotations from former faculty.

Many women who left academia mentioned that they did so due to a lack of response from institutional leadership to the devaluation they experienced. For example, as a white woman who left academia as a tenured psychology professor reported,

> I did really well in academia; I had publications in top-tier journals, received several federal grants, and had high teaching scores. I was well liked by my colleagues and had good relationships with all of them. The main reason I left is because I was [harassed] by a group of graduate students. They told lies about me (that I could counter with evidence) and spread false rumors. They questioned my competence, treated me rudely, and turned others against me. I am convinced they would not have treated me this way if I had been a man. It was completely damaging to my mental and physical health. What made it especially horrible is that I felt I did not get adequate support from my unit. In essence, they did nothing. It was one of the most painful times in my life...I now work in industry, make really good money, feel valued by my colleagues, and feel I am making an impact in the world. Leaving academia is one of the best decisions I have ever made. I would never, ever, go back.

While men who retired very rarely reported doing so due to devaluation, it was more common for women to retire because of the accumulation of formal and informal devaluation that they had experienced over their career. As a white woman who retired as a tenured economics professor reported,

> I had to sue my university to get equitable pay. My discipline, economics, is bad for women and humans who care about other people. Selfishness is a virtue. Co-authored with a male. He got all the credit. I never thought I would retire this young but I just got sick of the old boy networks, microagressions, mansplaining, and interrupting in meetings. And I was a dean!

And similarly, women who retired often reported that the institution did not respond appropriately to instances of devaluation. As a woman of color who retired as a tenured engineering professor stated,

> My department chair was a toxic person who ruled with fear and intimidation. He was clearly biased against female faculty. Nothing was done about this by upper administration...He divided people into good and bad based on preconceived notions and made no effort to learn about faculty contributions over the course of their careers.

References

[1] Katie Spoon, Nicholas LaBerge, K Hunter Wapman, Sam Zhang, Allison C Morgan, Mirta Galesic, Bailey K Fosdick, Daniel B Larremore, and Aaron Clauset. Gender and retention patterns among us faculty. *Science Advances*, 9(42):eadi2205, 2023.

[2] K. Hunter Wapman, Sam Zhang, Aaron Clauset, and Daniel B. Larremore. Quantifying hierarchy and dynamics in US faculty hiring and retention. *Nature*, 610(7930):120–127, 2022.

[3] Caterina De Bacco, Daniel B. Larremore, and Cristopher Moore. A physical model for efficient ranking in networks. *Science Advances*, 4(7):eaar8260, 2018.

[4] Jennifer Dykema, John Stevenson, Lisa Klein, Yujin Kim, and Brendan Day. Effects of e-mailed versus mailed invitations and incentives on response rates, data quality, and costs in a web survey of university faculty. *Social Science Computer Review*, 31(3):359–370, 2013.

[5] Morgan M. Millar and Don A. Dillman. Improving response to web and mixed-mode surveys. *Public Opinion Quarterly*, 75(2):249–269, 2011.

[6] Ian Van Buskirk, Aaron Clauset, and Daniel B. Larremore. An open-source cultural consensus approach to name-based gender classification. *arXiv preprint arXiv:2208.01714*, 2022.

[7] American Psychological Association et al. A guide for supporting trans and gender diverse students. *Washington, DC: APA Association of Graduate Students (APAGS)*, 2019.

[8] Michael Burawoy. *Ethnography unbound: Power and resistance in the modern metropolis.* Univ of California Press, 1991.

[9] Kathy Charmaz and Richard G. Mitchell. Grounded theory in ethnography. *Handbook of Ethnography*, pages 160–174, 2001.

[10] Stefan Timmermans and Iddo Tavory. Theory construction in qualitative research: From grounded theory to abductive analysis. *Sociological Theory*, 30(3):167–186, 2012.

[11] Michèle Lamont. *How professors think: Inside the curious world of academic judgment.* Harvard University Press, 2009.

[12] Kosuke Imai, Luke Keele, and Dustin Tingley. A general approach to causal mediation analysis. *Psychological methods*, 15(4):309, 2010.

[13] Anna A Igolkina and Georgy Meshcheryakov. semopy: A python package for structural equation modeling. *Structural Equation Modeling: A Multidisciplinary Journal*, 27(6):952–963, 2020.
